# Detection and Quantification of Microparticles from Different Cellular Lineages Using Flow Cytometry. Evaluation of the Impact of Secreted Phospholipase A_2_ on Microparticle Assessment

**DOI:** 10.1371/journal.pone.0116812

**Published:** 2015-01-14

**Authors:** Matthieu Rousseau, Clemence Belleannee, Anne-Claire Duchez, Nathalie Cloutier, Tania Levesque, Frederic Jacques, Jean Perron, Peter A. Nigrovic, Melanie Dieude, Marie-Josee Hebert, Michael H. Gelb, Eric Boilard

**Affiliations:** 1 Centre de Recherche en Rhumatologie et Immunologie, Centre de Recherche du Centre Hospitalier Universitaire de Québec, Faculté de Médecine de l’Université Laval, Québec, QC, Canada; 2 Centre de Recherche du CHUQ and Département d’Obstétrique-Gynécologie, Faculté de Médecine, Université Laval, Québec, QC, Canada; 3 Centre Hospitalier Universitaire de Québec, Québec, Canada; 4 Division of Rheumatology, Immunology and Allergy, Brigham and Women’s Hospital, Harvard Medical School, Boston, MA, United States of America; 5 Division of Immunology, Boston Children’s Hospital, Harvard Medical School, Boston, MA, United States of America; 6 Centre hospitalier de l’Université de Montréal (CRCHUM), Montréal, QC, Canada; 7 Department of Chemistry, University of Washington, Seattle, WA, United States of America; University of Sydney, AUSTRALIA

## Abstract

Microparticles, also called microvesicles, are submicron extracellular vesicles produced by plasma membrane budding and shedding recognized as key actors in numerous physio(patho)logical processes. Since they can be released by virtually any cell lineages and are retrieved in biological fluids, microparticles appear as potent biomarkers. However, the small dimensions of microparticles and soluble factors present in body fluids can considerably impede their quantification. Here, flow cytometry with improved methodology for microparticle resolution was used to detect microparticles of human and mouse species generated from platelets, red blood cells, endothelial cells, apoptotic thymocytes and cells from the male reproductive tract. A family of soluble proteins, the secreted phospholipases A_2_ (sPLA_2_), comprises enzymes concomitantly expressed with microparticles in biological fluids and that catalyze the hydrolysis of membrane phospholipids. As sPLA_2_ can hydrolyze phosphatidylserine, a phospholipid frequently used to assess microparticles, and might even clear microparticles, we further considered the impact of relevant sPLA2 enzymes, sPLA2 group IIA, V and X, on microparticle quantification. We observed that if enriched in fluids, certain sPLA_2_ enzymes impair the quantification of microparticles depending on the species studied, the source of microparticles and the means of detection employed (surface phosphatidylserine or protein antigen detection). This study provides analytical considerations for appropriate interpretation of microparticle cytofluorometric measurements in biological samples containing sPLA2 enzymes.

## Introduction

Extracellular vesicles (EV) are small membrane vesicles derived from cells upon activation or apoptosis. The classification of EVs is mostly based on their size, composition and most importantly on their process of release. Exosomes (50–100nm in diameter) are stored in cells and are liberated by exocytosis of multivesicular bodies. Apoptotic bodies (1000–5000nm in diameter) and microparticles (100–1000nm in diameter) are produced by plasma membrane budding during apoptosis and cell activation, respectively [1].

The release of microparticles (MP) implicates an increase of intracellular calcium and rearrangement of the cytoskeleton [2]. During this process, membrane asymmetry is generally lost, leading to the exposure of phosphatidylserine (PS) normally present only in the inner leaflet of the membrane bilayer. Functionally, the exposed PS is implicated in the promotion of the coagulation cascade [2, 3] and rapid (<10 minutes to some hours) clearance of MPs in blood circulation [4, 5, 6], mostly through its recognition by lactadherin and developmental endothelial locus-1 (Del-1) [7, 8]. Intriguingly, MPs in blood and in the synovial fluid of patients with rheumatoid arthritis (RA), an autoimmune inflammatory disease affecting the joints, are frequently deprived of surface PS [9, 10, 11, 12]. However, how MPs maintain membrane asymmetry in these conditions remains to be elucidated.

Apart from PS, MPs also express surface antigens and transport cargo (e.g. mRNA, microRNA, proteins) [1, 13] that originate from the donor cell, suggesting that different MPs might play distinct functions depending on the cell they are derived from. To date, MPs have been described as key actors in intercellular communication, as effectors in thrombosis, immunology, inflammation, reproduction, atherosclerosis, autoimmune diseases and cancer [13, 14, 15, 16, 17]. Thus, their precise detection in diverse biological fluids is crucial for the development of biomarkers and the comprehension of their functional activities.

Flow cytometry is the most widely used approach for the detection of MPs. However, studies showed that MPs smaller than approximately 0.5µm in diameter were not efficiently resolved by these technologies [18, 19, 20]. Multiple smaller MPs can be in fact detected simultaneously and are erroneously considered as single MP [19]. This process, referred to as swarm or coincidence detection, remains useful for the detection of smaller MPs, but it biases MP quantification and leads to misinterpretations of observations based on MP multi-color labeling. High sensitivity flow cytometers have been developed [12, 21, 22, 23, 24, 25, 26, 27, 28] and provide sufficient size resolution for the identification of MP subtypes, such as MPs decorated with autoantibodies and those containing organelles [12, 22]. However, as with conventional flow cytometers, protein aggregates [29, 30] and potentially other factors present in biological samples may interfere in measurements performed with novel generation flow cytometers.

A family of small (≈14kDa) soluble proteins, the calcium-dependent secreted phospholipases A_2_ (sPLA_2_), comprises enzymes that catalyze the hydrolysis of membrane phospholipids at position *sn*-2, producing free fatty acids and lysophospholipids [31, 32]. Currently, 10 human sPLA_2_s (11 in mice) have been described and classified in different groups according to their sequence homology, structure and number and position of disulphide bounds [32]. The different sPLA_2_ enzymes play non-redundant physio- and pathological roles in dietary lipid digestion, reproduction, coagulation, anti-microbial defense, asthma, allergy, atherosclerosis, RA, and cancer [32]. sPLA_2_s from the groups IIA, V and X are among the most abundant and active enzymes [33] and present distinct substrate specificity, supporting the notion that they might not be isozymes [33, 34, 35, 36]. For instance, sPLA_2_ IIA (like most sPLA_2_s) shows a strong preference for anionic phospholipids like PS, whereas sPLA_2_ V and X are unique among sPLA_2_s as they display significant activity on anionic and zwitterionic phospholipids, such as those expressed on surface of intact cells [31, 32, 33]. Consistent with their respective preference for anionic vs zwitterionic phospholipids, sPLA_2_ V and X, and not sPLA_2_ IIA, can efficiently release fatty acids from the plasma membrane of intact cells [32]. sPLA_2_ IIA, on the other hand, is highly potent at hydrolyzing Gram^+^ bacteria [31, 32] (rich in surface phosphatidylglycerol) and can utilize MPs produced by red blood cell (RBC), platelets and whole blood cells as substrate [22, 37]. As these enzymes are secreted, they can also interact with cells through binding to receptors, proteoglycans or other binding proteins [38, 39, 40]. Thus, there exist different sPLA_2_ enzymes, and their functions might be dictated by their substrate specificity, interaction with receptors and cellular/organ expression.

sPLA_2_s are secreted by numerous cellular lineages including platelets, neutrophils, macrophages, endothelial cells and fibroblasts [31, 32, 41]. sPLA_2_s and MPs are detected in most, if not all, biological fluids, including blood/plasma, bronchoalveolar lavages, cerebrospinal fluid, saliva, semen, synovial fluid, tears and urine, suggesting that a potential interaction between MPs and sPLA_2_s might occur *in vivo* [14, 16, 17, 42, 43, 44, 45, 46, 47, 48, 49, 50, 51]. MP quantification generally involves probes that recognize surface PS (such as annexin-V) and surface cell lineage antigens (using fluorochrome-conjugated antibodies). Accordingly, it was suggested that annexin-V probes were inappropriate for the detection of MPs in sPLA_2_s-containing fluids [52], and several authors also suggested that sPLA_2_s could clear MPs [15, 16, 51, 53, 54, 55, 56, 57, 58]. However, whether exposition of MPs to sPLA_2_s promotes MP clearance or impacts PS recognition by annexin-V probes has never been formally assessed.

In the present study, we aimed to determine the impact of some of the most abundant, and thereby the best-described sPLA_2_s, namely sPLA_2_ group IIA, V and X, on MP quantification. Since murine and human sPLA_2_s might display different affinities toward MPs, we used recombinant murine and human sPLA_2_s and conducted mechanistic investigations by using inactive sPLA_2_ mutants. Similarly, we surmised that, depending on their cellular source, MPs might harbor distinct components (lipids and receptors) with different affinities for sPLA_2_s, which might impact hydrolysis. Thus, we used MPs from platelets, RBC, endothelial cells, apoptotic thymocytes and cells from the male reproductive tract, all from human and mouse species, for our analyses. Using the most advanced flow cytometric methods for the detection and quantification of MPs, we made the observation that sPLA_2_ enzymes utilize MPs as substrate, and thereby impair the quantification of MPs differently depending on 1) the sPLA_2_ group implicated, 2) the species studied (human or mouse), 3) the cellular source of MPs and 4) the means of detection employed (annexin-V or antigen recognition by antibodies).

Our study provides comprehensive considerations for the detection of MPs of different cellular lineages using high sensitivity flow cytometry. Moreover, considering that MPs and sPLA_2_s are often concomitantly present in biological fluids and cells culture supernatants, our observations suggest that the modulation of certain types of MPs should be interpreted with caution, especially if sPLA_2_s are overexpressed, and might provide mechanistic insights on the contribution of sPLA_2_ in the biological functions of MPs.

## Materials and Methods

### Ethic statement

Human blood cells were obtained from citrated blood of healthy volunteers under an approved institutional review board protocol (Comité Éthique de la Recherche du CHU de Québec). The donors gave their written consent and this consent procedure was approved by the Comité Éthique de la Recherche du CHU de Québec.

Human thymuses from newborns and young children were obtained under an approved institutional review board protocol (Comité Éthique de la Recherche du CHU de Québec) following written consent (approved by the Comité Éthique de la Recherche du CHU de Québec) of the family after a cardiac surgery (CHU de Quebec).

Human epididymidal tissues from a 52-year-old donor were obtained through the transplantation program Québec Transplant (Quebec City, Canada) following written consents of the family. The donor had no known pathologies that could affect reproductive functions. Experiments were conducted according to the policies for the Human Studies with the approval of the ethical committee of the Institutional Review Board of the Centre Hospitalier Universitaire de Québec (CHUQ protocol 09.04.006).

Semen samples were obtained in our institution’s clinical andrology laboratory by masturbation from healthy volunteer donors under an approved institutional review board protocol (Comité Éthique de la Recherche du CHU de Québec). The donors gave their written consent, which was approved by the Comité Éthique de la Recherche du CHU de Québec.

In this study, Guidelines of the Canadian Council on Animal Care were followed in a protocol approved by the Animal Welfare committee at Laval University. Cardiac punctures were performed under isoflurane anesthesia, Thymus harvesting was performed after an isoflurane anesthesia followed by euthanasia with CO_2_ and all efforts were made to minimize suffering.

This study was reviewed and approved by our institutional review board (Comité Éthique de la Recherche du CHU de Québec) before its initiation.

### Production and Isolation of platelet MPs

Citrated blood was transferred to 50 ml tubes (BD Falcon) and was centrifuged 10 minutes at 282g (room temperature (RT)) without brake. The platelet-rich plasma was harvested and 1/5 volume of acid citrate dextrose (ACD) and 1/50 volume of EDTA were added. The PRP was then centrifuged 5 minutes at 400g (RT) and the supernatant was harvested and further centrifuged 5 minutes at 1300g (RT). The supernatant was discarded, the pellet was resuspended in 2ml of Tyrode’s buffer pH 6.5 and 13ml of Tyrode’s buffer pH 7.4 were added to the homogeneous preparation of platelets. The platelets were counted and diluted at 100x10^6^ platelets/ml with Tyrode’s buffer pH 7.4 containing 5mM of CaCl_2_. The platelets were stimulated 15 hours with 0.5µg/ml of collagen (collagen reagent Horm suspension from Nycomed), 2 hours with 0.5 Unit/ml of thrombin (from bovine serum, Sigma) or 1mg/ml of heat aggregated human IgG (HA-IgG) at RT. Human HA-IgG were obtained by incubating human IgG (Sigma) 1 hour at 63°C. Platelet activation was stopped by addition of 20mM of EDTA and the preparation was centrifuged 10 minutes at 2000g (RT) twice to eliminate remnant platelets. The supernatant was collected and centrifuged 90 minutes at 18 000g (18°c) using swinging buckets, the supernatant was discarded and the pellet (containing platelet MPs) was resuspended in 100µl of Tyrode’s buffer containing 5mM of CaCl_2_. MPs were conserved in aliquots at -80°c prior utilization. When fluorescent platelet MPs were required, human platelets were incubated in presence of 1µM cell tracker CMFDA (5-chloromethylfluorescein diacetate, Invitrogen, ON, Canada) for 15 minutes in the dark according to the manufacturer protocols, washed and then stimulated as described above.

Mouse platelets were obtained from blood of CD41-YFP mice from our animal housing facility [59]. Mouse blood was obtained by cardiac puncture in syringe (1ml 25g 5/8 from BD) preloaded with 200µl of ACD. The blood was then transferred to an eppendorf tube containing 350µl of Tyrode’s buffer pH 6.5. The blood was centrifuged 3 minutes at 600g (RT), the PRP was collected and centrifuged 2 minutes at 400g (RT). The PRP was conserved and centrifuged 5 minutes at 1300g (RT). The supernatant was discarded, the pellet (containing the platelets) was quickly resuspended with 500µl of Tyrode’s buffer pH 6.5 and 14.5ml of Tyrode’s buffer pH 7.4 were added. The platelets were counted and diluted at 100x10^6^ platelets/ml with Tyrode’s buffer pH 7.4 containing 5mM of CaCl_2_. The platelets were stimulated overnight with 0.5µg/ml of collagen (collagen reagent Horm suspension from Nycomed) at RT. Platelet activation was stopped by addition of 20mM of EDTA and the preparation was centrifuged twice 10 minutes at 2000g (RT) to eliminate remnant platelets. The supernatant was harvested and centrifuged 90 minutes at 18000g (18°c) using swinging buckets, the supernatant was discarded and the pellet (containing the platelet MPs) was resuspended in 100µl of Tyrode’s buffer containing 5mM of CaCl_2_. MPs were aliquoted and kept at -80°c prior utilization.

### Production and isolation of erythrocyte MPs

Citrated blood was transferred to 50 ml tubes (BD Falcon) and was centrifuged 10 minutes at 282g (room temperature (RT)) without brake to stop the centrifugation. The PRP and the buffy coat were eliminated, 200µl of the erythrocyte fraction was added in 50 ml of distilled water (filtered on 0.22µm) for 10 minutes, and then 5.5ml of PBS 10x (filtered on 0.22µm) were added to stop the hypotonic reaction. Mouse erythrocytes were obtained from blood of C57BL/6J purchased from the Jackson Laboratory. The blood was collected by a cardiac puncture in syringe (1ml 25g 5/8 from BD) containing 200µl of ACD and then transferred to an eppendorf tube containing 350µl of Tyrode’s buffer pH 6.5. For mouse erythrocytes, blood (from 2 mouse donors) in a 15 ml tube (BD Falcon) was centrifuged 10 minutes at 282g (RT), then the PRP and the buffy coat were eliminated. Production of mouse erythrocyte MPs was performed as for human erythrocytes. Supernatants containing MPs were aliquoted and frozen at -80°c.

### Production and isolation of endothelial cell MPs

HUVEC (Clonetic, San diego, CA) and EOMA (ATCC CRL-2586) endothelial cells were cultured in complete medium until confluence. HUVEC were cultured in EGM-2MV complete medium (Clonetics) and EOMA cells were cultured in DMEM containing 10% of FBS. The cells were washed twice in serum-free medium and then incubated 15 minutes at 37°c with pre-warmed serum-free medium containing 1µM CMFDA. After 15 minutes, the dye working solution was removed and the cells washed twice with serum-free medium. Then, the cells were incubated 24h at 37°c with complete medium containing 10ng/ml of TNFα (R&D system; 210-TA). The supernatants were collected and centrifuged 10 minutes at 800g to eliminate cells and apoptotic bodies. Supernatants containing MPs were aliquoted and frozen at -80°c.

### Production and isolation of thymocyte MPs

Human thymuses from newborns and young children were used as a source of thymocytes. Mouse thymuses were obtained from 4–6 week old C57BL/6J mice. Human and mouse thymuses were crushed through 70 µm nylon cell strainer (BD Falcon) and thymocytes were collected in RPMI 1640 (Wisent). Before seeding, human and mouse thymocytes were incubated in presence of 1µM cell tracker CMFDA in RPMI without FBS for 15 minutes in the dark and washed twice with PBS 1X. Human and mouse thymocytes were seeded at 5.10^6^ cells/ml in 12 ml of RPMI 1640 (Wisent) with 5% FBS in 25 cm^2^ flasks (BD Falcon). After 24 hours, supernatants were collected and centrifuged 10 minutes at 800g to eliminate cells, debris and larger apoptotic bodies. Supernatant containing MPs were then collected, aliquoted and kept at -80°c.

### Preparation of epididymosomes

Human epididymides were removed under artificial circulation to preserve organ assigned for transplantation and tissue’s integrity. Mouse epididymides were obtained from six C57BL/6J mice (8 to 10 week old). Epididymides were kept on ice until harvest of epididymal fluid containing extracellular vesicles, these latest being referred to as epididymosomes [17]. Intraluminal perfusion technique was adapted as already described [60]. In brief, after removal of the connective tissues from the middle cauda epididymidis, a small incision was made to allow the insertion of a catheter into the isolated tubule. The lumen of the tubule was perfused with PBS (137 mM NaCl, 3 mM KCl, 8 mM Na2HPO4, and 1.5 mM KH2PO4) at a rate of 10 µl/min under the control of a syringe pump. The perfusate was collected through the vas deferens until a clear, sperm-free fluid was obtained. Epididymal fluid was separated from spermatozoa by centrifugation at 700 x g for 10 min at 4°C. Supernatant was further centrifuged twice at 3000 x g for 20 min at 4°C to remove cellular debris. Final supernatant was stored at -80°C until flow cytometry analysis.

### Preparation of prostasomes

Semen samples were obtained by masturbation from healthy volunteer donors. Between two and five days of sexual abstinence were required before semen collection. A pool of three semen samples that met the World Health Organization’s reference values for semen analysis were included in the study. Once collected, samples were liquefied at room temperature and seminal plasma was separated from spermatozoa by centrifugation at 800 x g for 10 min at 4°C. Seminal plasma was further centrifuged twice at 3000 x g for 20 min at 4°C and supernatant containing prostasomes was frozen at -80°C until flow cytometry analysis.

### Human synovial fluids analysis

Human knee synovial fluids from confirmed RA patients were obtained and analyzed by time-resolved fluorescence immunoassays to determine the concentration of the sPLA_2_s, as already described [43].

### Reagents and antibodies

Fluorescent Sky Blue polystyrene microspheres were obtained from Spherotech (IL, USA). Various sizes were used: 0.04 to 0.09 µm (mean, 0.09 µm), 0.4 to 0.6 µm (mean, 0,45 µm), 0.7 to 0.9 (mean, 0.84 µm), 2.5 to 4.5 µm (mean, 3.2 µm). Yellow-green FluoSpheres carboxylate-modified microspheres of 1µm were obtained from Invitrogen Molecular Probes (Oregon, USA). To process the data quantitatively, polystyrene microsphere (15µm diameter; Polysciences, PA, USA) were added to each tubes.

### Incubation of MPs with sPLA_2_ enzymes

Mouse and human MPs (5x10^6^, quantified by flow cytometry on the basis of annexin-V binding) were incubated in 100µl of PBS 1X (filtered on 0.22µm pore size membrane (Fisher Scientific, ON, Canada)) containing 0.1 and 1µg/ml of mouse or human recombinant sPLA_2_ IIA, V, X, mouse inactive mutant sPLA_2_ X H48Q or human inactive mutant sPLA_2_ V H48Q at 37°c for 1 and 6 hours. Control samples (without sPLA_2_) were also incubated 1 or 6 hours at 37°c. When sPLA_2_ activity endogenously expressed in mouse plasma was assayed, 5x10^6^ human CMFDA^+^ platelet MPs were incubated in 100µl of plasma free platelets (PFP) from C57BL6 or transgenic mice expressing the human sPLA_2_ IIA [61] at 37°c for 6 hours.

### Platelet free plasma preparation

Mouse blood (from C57BL6 or transgenic mice expressing human sPLA_2_ IIA) was obtained by cardiac puncture in syringe (1ml 25g 5/8 from BD) preloaded with 200µl of ACD. The blood was then transferred to an eppendorf tube containing 350µl of Tyrode’s buffer pH 6.5 and centrifuged 20 minutes at 2500g. The platelet poor plasma was collected and centrifuged 2 minutes at 13000g. The PFP was collected and stored at -80°c.

### MP labeling

After incubation with sPLA_2_, fluorescent probe-conjugated annexin-V and antibodies directed against surface markers (all from BD Biosciences) were used to label MPs. Antibodies and annexin-V were incubated 30 minutes with MPs at RT. PE rat anti-mouse CD4 (clone RM4–5) was used at 4ng/µl, APC rat anti-mouse CD31 (clone MEC 13.3) was used at 4ng/µl, PE rat anti-mouse CD41 (clone MWReg 30) was used at 1ng/µl, APC rat anti-mouse TER-119/erythroid cells (clone TER-119) was used at 2ng/µl, PE-Cy7 mouse anti-human CD3 (clone SK7) was used at 4ng/µl, Alexa-fluor 647 mouse anti-human CD31 (clone WM59) was diluted 1/50, APC mouse anti-human CD41a (clone HIP8) was diluted 1/50, FITC mouse anti-human CD235a (clone GA-R2 (HIR2)) was used at 5ng/µl, Brillant Violet 421 mouse anti-human CD13 (clone WM15) was diluted 1/50. FITC-, APC- and V450- annexin-V were diluted 1/50. For all conditions, more than 5,000 annexin-V^+^ MPs were acquired in control samples (i.e. without sPLA_2_).

### Flow cytometry analyses

All the buffers were filtered on a 0.22µm pore size membrane (Fisher Scientific, ON, Canada). A forward scatter (FSC) coupled to a photomultiplier tube (PMT) ‘small particles option’ (FSC-PMT) (rather than the usual diode [23]) with a 488nm solid state, 100mW output blue laser (rather than the conventional 20 mW), a 633 nm HeNe, 20mW output red laser and a 405nm solid state diode, 50mW output violet laser were mounted on the FACS Canto II Special Order Research Product used for this study (BD Biosciences, ON, Canada). The high sensitivity flow cytometer is equipped with FSC-PMT and a Fourier optical transformation unit reducing the background noise and increasing the angle of diffusion to 25.8° (not 9° as for conventional FSC diode). All these improvements allow the detection of smaller particles. Flow cytometer performance tracking was performed daily (before all analyzes) using the BD cytometer setup and tracking beads (BD Biosciences, San Jose, CA, USA) to monitor the constant performance over time. The chosen parameters were optimal to detect microspheres from 100 to 1000 nm simultaneously on the FSC-PMT. The fluorescence was used as trigger signal and the positive fluorescent events were portrayed in a SSC/FSC-PMT graph. The MP gate of detection was designed according to the acquisition of Sky blue and yellow-green microspheres of mean diameter of 90, 450, 840, 1000 and 3200 nm. For SSC, the assigned voltage was 407 Volts and the threshold was 200. For FSC-PMT, the assigned voltage was 363 Volts and the threshold was 0. MPs were acquired at low speed at a rate of approximately 10µl/min. Each antibody was incubated in PBS 1X alone, in absence of MP preparation or sample, and acquired to determine the background noise, if any.

### Recombinant phospholipase A_2_ enzymes

Human and mouse recombinant enzymes were produced as already described [33].

### Statistical analyses

All data are mean ± SEM. Statistical significance between 2 groups was determined using unpaired Student *t* tests. All the statistical analyses were performed using Prism software 4.00 (GraphPad Software, CA, USA).

## Results

### Optimization of high sensitivity flow cytometric methods for the detection of MPs

We focused our study on EVs that could be reliably detected by high sensitivity flow cytometry. MPs are widely described as EVs ranging from 100 to 1000 nm, expressing PS, and exposing specific surface markers originating from their cellular origin. Using these properties, we can detect MPs cytofluorometrically using annexin-V (which recognizes PS) and specific antibodies against surface markers directly coupled to fluorochromes. Furthermore, fluorescent MPs can be produced using CellTracker 5-chloromethylfluorescein diacetate (CMFDA), a probe that freely passes through cellular membrane and subsequently converted to a fluorescent cell-impermeant product by cytosolic esterases. By activating cells pre-loaded with this reagent, the fluorescence is encapsulated within MPs, permitting their detection [12]. Rather than using CMFDA as intracellular fluorescent marker for mouse platelets, we used platelets isolated from yellow fluorescent protein (YFP)-CD41 transgenic mice [59]. In these mice, platelets express YFP, which is present within MPs. We thus verified the impact of sPLA_2_s on these various types of MP labelling.

Due to the small size of MPs, conventional flow cytometry is not optimal for the detection and quantification of MPs. However, most recent improvements in this technology have significantly enhanced the investigators’ capabilities to detect and more efficiently quantify MPs [12, 22, 23, 27, 28]. In our studies, we used a high sensitivity flow cytometer *small particle option* equipped with a more powerful blue laser (100mW rather than 20mW) and a Fourier bar that provides lower background and noise and increases the angle of diffusion and a photomultiplier tube (PMT) coupled to the forward scatter (FSC)[12, 21, 22].

**Figure 1:**
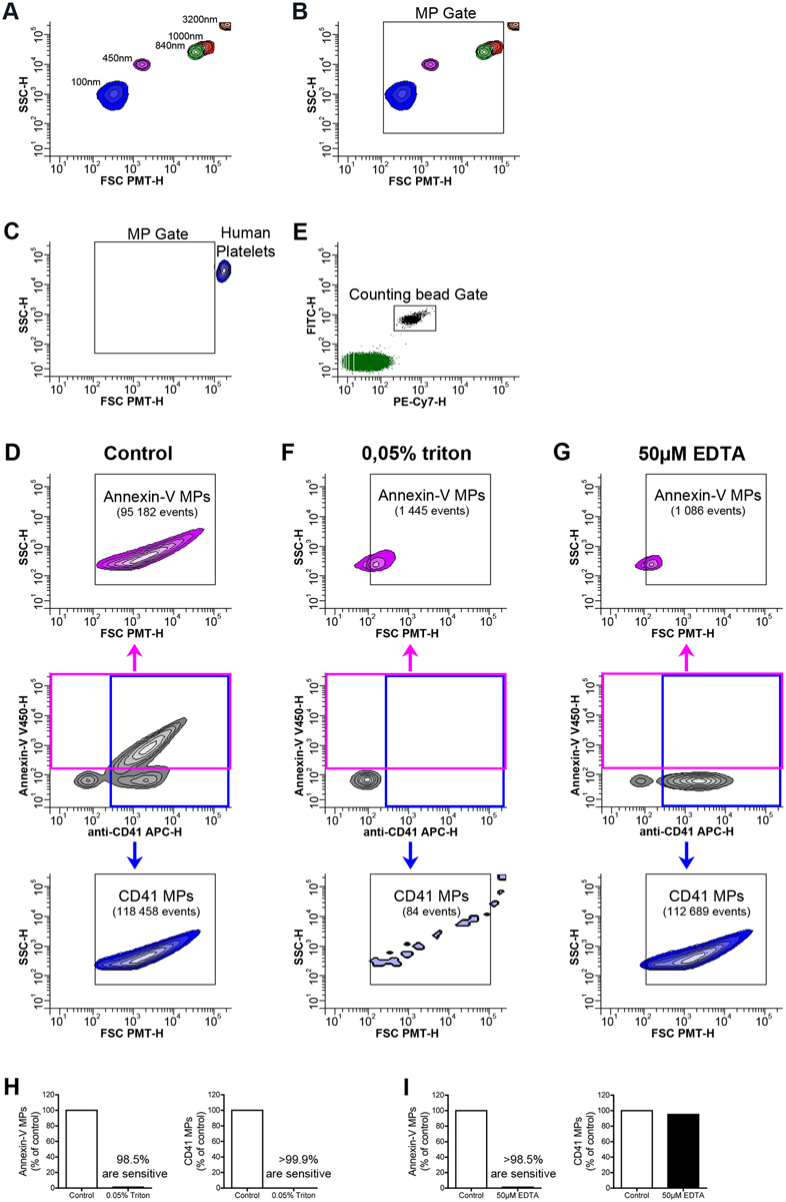
Optimization of flow cytometric methods for the detection of MPs. (A, B) Acquisition of fluorescent microspheres of 100nm (Blue), 450nm (pink), 840nm (green), 1000nm (red), 3200nm (orange) in diameter on a flow cytometer Canto II modified with a FSC-PMT small particles option. (B) A MP gate including particles from 100 to 1000nm in diameter based on the microsphere sizes (FSC-PMT-H) is presented and used to detect MPs. (C) Portrayal of relative size of human platelets detected with fluorochrome-conjugated antibodies directed against CD41. (D) FSC-PMT/SSC portrayal of platelet MPs detected with annexin-V and fluorochrome-conjugated antibodies directed against CD41 in absence of treatment (control). (E) A known concentration of auto-fluorescent polystyrene microspheres (15 µm in diameter) was added in each tube and a determined number of beads was acquired in the counting bead gate to quantitatively process the data. (F, G) FSC-PMT/SSC portrayal of platelet MPs detected with annexin-V and fluorochrome-conjugated antibodies directed against CD41 and treated with 0.05% triton (F) and 50µM EDTA (G). Total annexin-V^+^ events are detected in the pink gate (middle panel) and the quantity of annexin-V^+^ MPs is determined in the Annexin-V MP gate (upper panel). Total CD41^+^ events are detected in the blue gate (middle panel) and the quantity of CD41^+^ MPs is determined in the CD41 MP gate (lower panel). Data are representative of 5 independent experiments. (H) Triton sensitivity of the platelet MPs detected using fluorochrome-conjugated annexin-V (left panel) and fluorochrome-conjugated antibodies directed against CD41 (right panel) is presented as % of untreated (control). (I) EDTA sensitivity of annexin-V (left panel) and CD41 (right panel) labeling is presented as % of untreated (control). Data are representative of 5 independent experiments.

In a first set of experiments, we optimized the settings of the flow cytometer using fluorescent microspheres of defined dimensions to create a gate (MP gate) in which particles with relative dimensions ranging from approximately 100 to 1000 nm are expected (Fig. 1A,B). As microspheres have a different refractive index than biological MPs and cells [18, 19], they may behave differently depending on the type of flow cytometer used and its optical units. Thus, for the setting of the upper limit of the MP gate, we also included a preparation of unactivated platelets (*i.e.* not containing MPs) (Fig. 1C). We found that we could resolve all the tested microspheres (Fig. 1A,B). Consistent with the expected diameter of human platelets (mean diameter between 2 and 5µm), platelets were detected similarly to the 3.2µm microspheres, and were readily distinguishable from the 1µm microspheres and those smaller (Fig. 1C). While factors such as size, shape, surface roughness, granularity and angle of collection affect light scattering, scatter intensity of smaller particles, especially those smaller in diameter than the wavelength of light (here 488nm), is greatly dependent on the refractive index [18]. The lower limit of the MP gate was thus arbitrary set, based on the detection of the smaller microspheres and recognizing the limitations of this approach. As expected, high sensitivity flow cytometry also efficiently detected annexin-V^+^-, CD41^+^-, CMFDA^+^—and YFP^+^-MPs (Fig. 1D and S1 Fig). Notably, all the platelet MPs (positive for annexin-V, CD41, CMFDA and YFP) were contained within the designed MP gate (Fig. 1D and S1 Fig.).

To confirm that the MPs contained within the MP gate were genuine MPs and not protein aggregates (formed by complexes of annexin-V or fluorochrome-conjugated antibodies), we verified the MP membrane sensitivity to detergent using a well-reported assay [29]. In this test, the membrane moiety of MPs is dissolved by TritonX-100 while protein aggregates remain intact [9, 12, 29]. Since annexin-V recognizes PS in a calcium dependent manner, we further confirmed its specific recognition of MPs by chelating Ca^2+^ ions. Using these optimized methods to detect MPs after adding a known number of fluorescent polystyrene microspheres (15 µm in diameter) to each tube to quantitatively process the data (Fig. 1E), we demonstrate the specificity of our measurements as more than 98% of the MPs detected using annexin-V or an antibody against CD41 were eliminated by detergent treatment (Fig. 1D, F, H). Moreover, annexin-V failed to recognize MPs in presence of 50µM EDTA (Fig. 1D, G, I). Using the same conditions, we verified our capabilities to detect MPs from RBC, endothelial cells, apoptotic thymocytes and from epithelial cells of the male reproductive tract (see S2–S5 Figs.).

**Figure 2:**
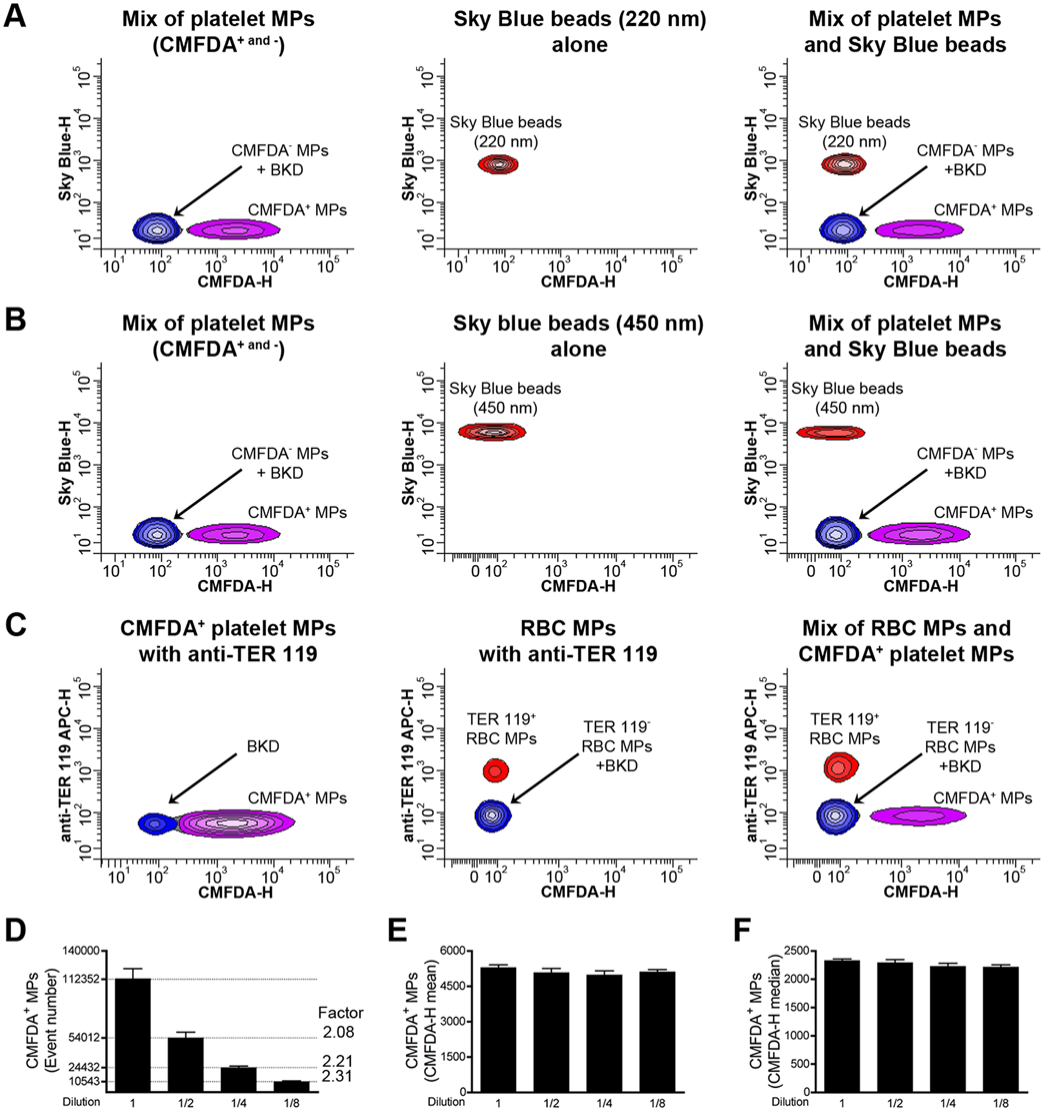
Study of swarm detection in high sensitivity flow cytometry. (A) A mixture of CMFDA^-^ and CMFDA^+^ platelet MPs (CMFDA^- and +^) and sky blue beads (220 nm in diameter) were analyzed alone (left and middle panel respectively) or mixed (right panel) and their detection resolved on the basis of fluorescence. (B) CMFDA^- and +^ platelet MPs and sky blue beads (450 nm in diameter) were analyzed alone (left and middle panel respectively) or mixed (right panel) prior to detection on the basis of fluorescence. (C) CMFDA^+^ platelet MPs and RBC MPs labeled with antibodies directed against TER 119 are analyzed alone (left and middle panel respectively) or mixed (right panel). (D, E, F) CMFDA^+^ platelet MPs were diluted serially thrice (2-fold dilution) and analyzed by high sensitivity flow cytometry to determine their concentration (D), the CMFDA-height (H) mean of fluorescence (E) and the CMFDA-H median of fluorescence (F) are presented. Data are mean ± SEM of 5 independent experiments. BKD = Background noise.

Coincidental detection of multiple MPs (called swarm detection) allows detection of smaller MPs when present at sufficient concentration, but compromises their accurate quantification and prohibits usage of simultaneous multi-marker labeling [19, 62]. To determine the involvement of swarm detection in our experimental conditions, we analyzed a mixture of fluorescent microspheres (sky blue, 0.22 and 0.45µm in diameter) and green fluorescent CMFDA^+^ platelet MPs (Fig. 2A–B). We found that both types of microspheres were readily distinguishable from green fluorescent MPs, and that no MPs displayed both labels simultaneously. Furthermore, platelet MPs labeled with a fluorescent green dye were efficiently resolved and discriminated from RBC MPs labeled with an anti-TER 119 antibody (Fig. 2C) confirming the absence of significant coincidence detection in our cytofluorometric conditions.

Next, to validate our quantitative strategies, we performed serial dilutions of CMFDA-labelled platelet MPs and determined their concentration and mean fluorescence intensity (MFI). In principle, if swarm detection does not interfere in cytofluorometric measurements, MP concentrations should be reduced accordingly to dilution factors while the MFI should remain constant. Notably, MP concentrations were consistently reduced with their respective dilution factors although they expressed constant fluorescence intensity (Fig. 2 D–F).

### Impact of sPLA_2_s on platelet MPs

Having confirmed our methodological approaches for the quantification of MPs, we aimed to determine the impact of soluble factors present in biological fluids potentially interfering in MP detection. Platelet MPs are the most abundant MPs circulating in the bloodstream and are involved in many physiological and pathological processes [9]. Since platelet MPs and sPLA_2_s are present simultaneously in blood circulation, this prompted us to determine if sPLA_2_ enzymes might affect their detection and quantification.

**Figure 3:**
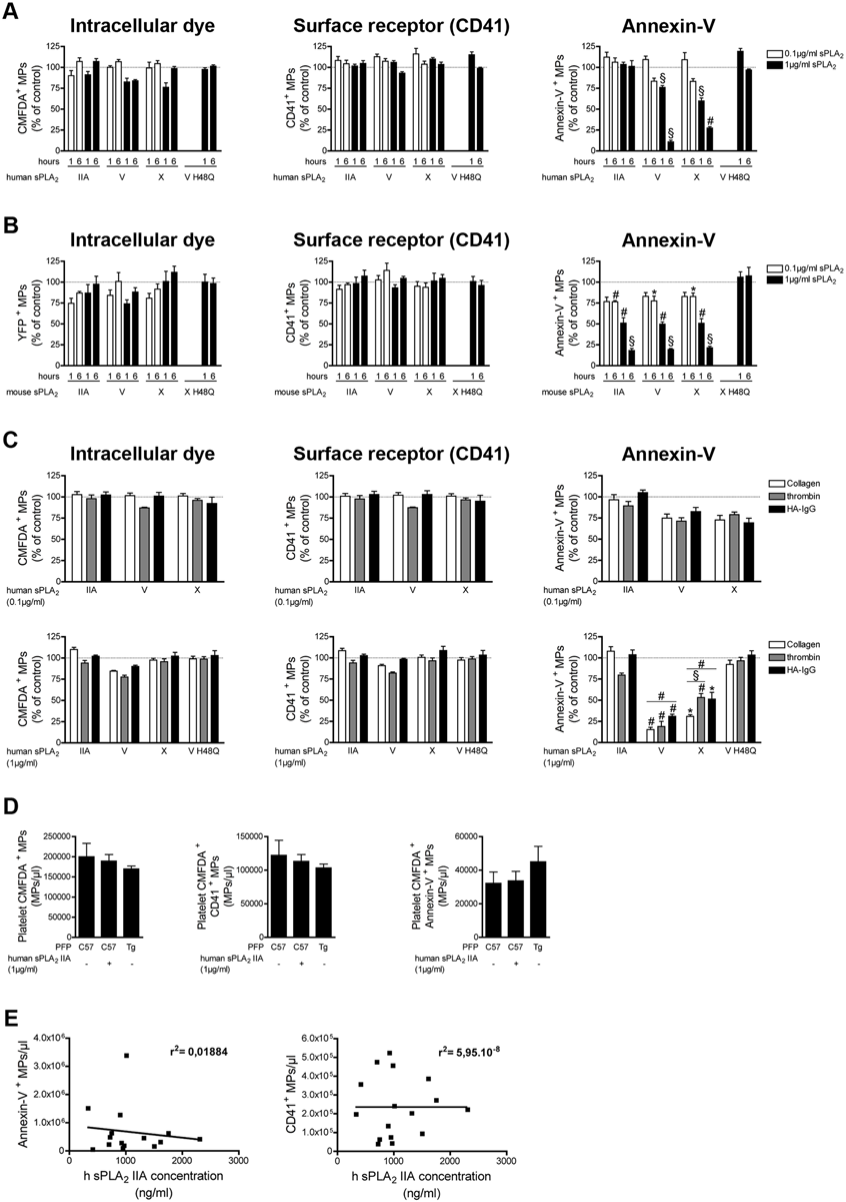
Impact of human and mouse sPLA_2_s on platelet MPs. (A) MPs from human platelets (stimulated with collagen) labeled with the CMFDA cell tracker were incubated for 1 and 6 hours at 37°c in absence or in presence of indicated concentrations of human recombinant sPLA_2_ IIA, V, X, or 1µg/ml of the inactive mutant V H48Q. Fluorochrome-conjugated antibodies directed against CD41 and fluorochrome-conjugated annexin-V were used to assess the quantities of CMFDA^+^ MPs (left panel), of CD41^+^ MPs (middle panel), of annexin-V^+^ MPs (right panel) and were compared to the untreated conditions (dotted line). Data are mean ± SEM of 5 independent experiments presented as % of untreated (control) (B) MPs from mouse platelets (stimulated with collagen), identified using YFP as fluorescent tracker, were incubated 1 and 6 hours at 37°c, in absence or in presence of indicated concentrations of mouse recombinant sPLA_2_ IIA, V, X, or 1µg/ml of the inactive mutant X H48Q. Fluorochrome-conjugated antibodies directed against CD41 and fluorochrome-conjugated annexin-V were used to determine the concentrations of YFP^+^ MPs (left panel), of CD41^+^ MPs (middle panel), of annexin-V^+^ MPs (right panel) and then compared to the untreated conditions (dotted line). Data are mean ± SEM of 5 independent experiments presented as % of untreated (control). (C) MPs from human platelets labeled with the CMFDA cell tracker and obtained following stimulation with collagen, thrombin or HA-IgG were incubated 6 hours at 37°c in absence or in presence of indicated concentration of human recombinant sPLA_2_ IIA, V and X and 1µg/ml of the inactive mutant sPLA_2_ V H48Q. Fluorochrome-conjugated antibodies directed against CD41 and fluorochrome-conjugated annexin-V were used to assess the quantities of CMFDA^+^ MPs (left panel), of CD41^+^ MPs (middle panel), of annexin-V^+^ MPs (right panel) and then compared to the untreated conditions (dotted line). Data are mean ± SEM of 3 independent experiments presented as % of untreated (control). (D) MPs from human platelets (stimulated with collagen) labeled with the CMFDA cell tracker were incubated 6 hours at 37°c in PFP of C57BL6 (supplemented or not with 1µg/ml of recombinant human sPLA_2_ IIA) or transgenic mice expressing the human sPLA_2_ IIA (Tg). Fluorochrome-conjugated antibodies directed against CD41 and fluorochrome-conjugated annexin-V were used to assess the quantities of CMFDA^+^ MPs (left panel), of CMFDA^+^ CD41^+^ MPs (middle panel) and CMFDA^+^ annexin-V^+^ MPs (right panel). Data are mean ± SEM of 3 independent experiments. (E) Concentrations of Annexin-V^+^ MPs and CD41^+^ MPs present in the synovial fluids of RA patients determined by high sensitivity flow cytometry and correlated to the concentration of human sPLA_2_ IIA assayed (in the same synovial fluids) by time-resolved immunofluorescence analysis. * P< .05; # P< .01; § P< .001.

MPs derived from human and mouse platelets were incubated with various concentrations of sPLA_2_ IIA, V and X of human and mouse species, respectively. While none of the sPLA_2_s tested could affect the detection of MPs when an intracellular tracker (CMFDA or YFP) or antibodies against CD41 were used (Fig. 3A–B left and middle panel), we observed that human and mouse sPLA_2_ V and X dose and time dependently reduced the number of annexin-V^+^ MPs (**Fig. 3A–B right panel**). Surprisingly, whereas mouse sPLA_2_ IIA efficiently decreased the number of annexin-V^+^ MPs, human sPLA_2_ IIA had no significant impact (**Fig. 3A–B right panel**). Since human sPLA_2_ IIA efficiently hydrolyzed membranes from *E. coli* (**S6 Fig**), this confirmed that the discrepancy between the action of murine and human sPLA_2_ IIA was not due to incorrect folding of the recombinant human enzyme. Thus, all the sPLA_2_s enzymes tested, except human sPLA_2_ IIA, impact the recognition of platelet MPs by annexin-V, but none of them clear platelet MPs.

To determine whether this action was due to PS hydrolysis or membrane masking by sPLA_2_s, platelet MPs were incubated with inactive mutant sPLA_2_s (sPLA_2_ V H48Q and sPLA_2_ X H48Q) [63]. No decrease of annexin-V^+^ MPs was observed in presence of the inactive mutants, suggesting that sPLA_2_s impede the detection of MPs by annexin-V probes through PS hydrolysis (**Fig. 3A–B right panel**).

Depending on the platelet trigger, platelet MPs express distinct platelet content [64]. This prompted us to verify whether sPLA_2_s could impact platelet MP detection differently depending on the stimuli implicated. We thus incubated human platelet MPs generated under 3 types of stimulation (*i.e.* collagen, thrombin and heat aggregated-IgG (HA-IgG)) and verified their susceptibility to human sPLA_2_ enzymes. Although each type of platelet MPs were equally poorly susceptible to sPLA_2_ enzymes when present at low concentration (0.1 µg/ml), higher concentrations of sPLA_2_ V and X revealed their modest, but significant, preference for collagen-induced platelet MPs compared to MPs produced using thrombin and HA-IgG (**Fig. 3C**). Thus, although the platelet activation pathway only modestly affects the impact of sPLA_2_ V and X on annexin-V labelling, it did not alter sPLA_2_ IIA impact on platelet MP detection.

We next investigated whether the presence of plasma might influence the activity of sPLA_2_ IIA toward platelet MPs, thereby affecting their detection. The platelet-free plasma (PFP) obtained from transgenic mice overexpressing human sPLA_2_ IIA (>1µg/ml in serum) [65] was used for these experiments and we included the PFP from C57BL6 mice (which naturally lack sPLA_2_ IIA) [66] as control. We observed that the expression of annexin-V and CD41 markers on CMFDA-labeled platelet MPs incubated in plasma of C57BL6 and transgenic sPLA_2_ IIA mice remained constant, even when exogenous recombinant human sPLA_2_ IIA was added to the test tubes (**Fig. 3D**), suggesting that plasma does not influence the impact of sPLA_2_ IIA on detection of platelet MPs.

MPs, especially those of platelet origin, and sPLA_2_ IIA (average 1µg/ml, and up to 2.3 µg/ml) are abundant in the synovial fluid of patients with RA [14, 43]. sPLA_2_s V and X are also present but at lower levels (approximately 11.1ng/ml and 0.36ng/ml, respectively) [43]. While annexin-V is frequently used to detect MPs in RA synovial fluid, we measured the concentrations of platelet MPs and of sPLA_2_ IIA present simultaneously in RA patients. Consistent with our findings made *in vitro*, we observed no significant correlation (negative nor positive) between the concentrations of human sPLA_2_ IIA and MPs (**Fig. 3E**). Thus, our *ex vivo* experiments confirm that human platelet MPs can be detected successfully in biological fluids, even in presence of high levels of human sPLA_2_ IIA.

### Impact of sPLA_2_s on MPs from erythrocytes

Erythrocytes are the most abundant cellular lineage present in blood where they generate MPs, possibly to eliminate modified antigens and to prevent the exposure of dangerous molecules [67]. After platelet MPs, erythrocyte-derived MPs are the second most abundant in blood circulation [9]. Thus, we aimed to evaluate the action of sPLA_2_s on erythrocyte-derived MPs.

**Figure 4:**
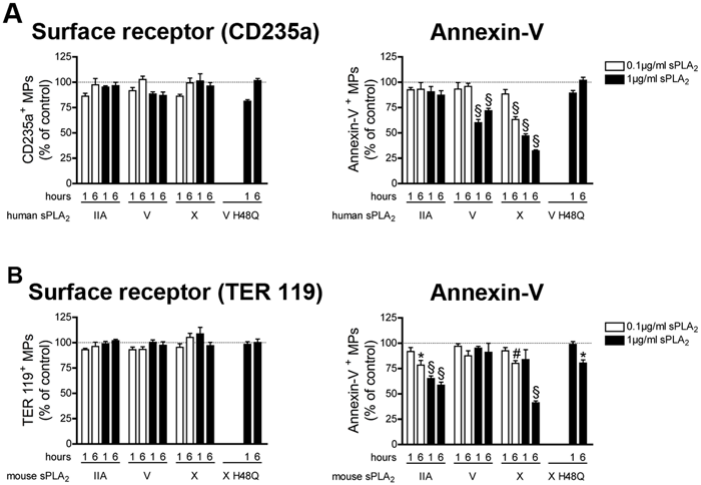
Impact of human and mouse sPLA_2_s on erythrocyte MPs. (A) MPs from human erythrocytes were incubated for 1 and 6 hours at 37°c in absence or in presence of indicated concentrations of human recombinant sPLA_2_ IIA, V, X, or 1µg/ml of the inactive mutant V H48Q. Fluorochrome-conjugated antibodies directed against CD235a and fluorochrome-conjugated annexin-V were used to assess the quantities of CD235a^+^ MPs (left panel), of annexin-V^+^ MPs (right panel) and were compared to the untreated conditions (dotted line). Data are mean ± SEM of 5 independent experiments presented as % of untreated (control) (B) MPs from mouse erythrocytes were incubated 1 and 6 hours at 37°c, in absence or in presence of indicated concentrations of mouse recombinant sPLA_2_ IIA, V, X, or 1µg/ml of the inactive mutant X H48Q. Fluorochrome-conjugated antibodies directed against TER 119 and fluorochrome-conjugated annexin-V were used to determine the concentrations of TER 119^+^ MPs (left panel) and annexin-V^+^ MPs (right panel) and then compared to the untreated conditions (dotted line). Data are mean ± SEM of 5 independent experiments presented as % of untreated (control). * P< .05; # P< .01; § P< .001.

Human and mouse erythrocyte-derived MPs were incubated with human and mouse sPLA_2_s, respectively. We observed that none of sPLA_2_s could impact the detection of erythrocyte MPs when antibodies against CD235a (human) or TER119 (mouse) were used (**Fig. 4A–B left panel**). However, human sPLA_2_ V and X (but not IIA) and mouse sPLA_2_ IIA and X (but not V), induced a time and concentration dependent reduction of annexin-V^+^ MPs, which occurred through PS hydrolysis since inactive mutants failed to induce this decrease (**Fig. 4A–B right panel**). Thus, mouse sPLA_2_ IIA, human sPLA_2_ V and sPLA_2_ X (human and mouse) hydrolyze the PS exposed on erythrocytes MPs, but none of them consume erythrocyte MPs.

### Impact of sPLA_2_s on MPs from endothelial cells

Endothelial cells are perfectly localized to release MPs in the bloodstream under physiological conditions or in pathologies such as atherosclerosis [68, 69]. Furthermore, sPLA_2_ IIA, V and X contribute in atherosclerosis, and endothelial cells constitutively express sPLA_2_ V [31, 32, 70]. As endothelial cell MPs and sPLA_2_ are simultaneously present in blood, we examined the action of sPLA_2_s on endothelial cell MPs.

**Figure 5:**
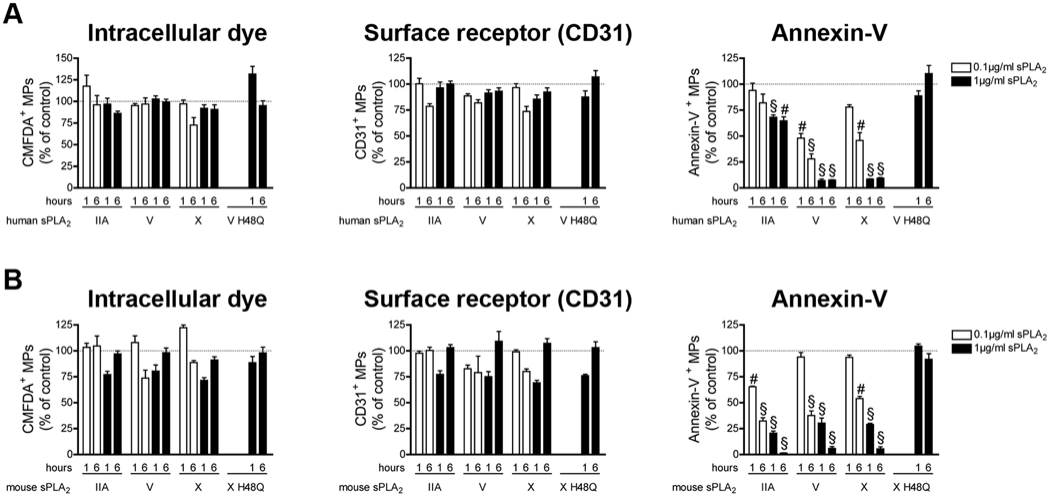
Impact of human and mouse sPLA_2_s on endothelial cell MPs. (A) MPs from HUVEC labeled with the CMFDA cell tracker were incubated for 1 and 6 hours at 37°c in absence or in presence of indicated concentrations of human recombinant sPLA_2_ IIA, V, X, or 1µg/ml of the inactive mutant V H48Q. Fluorochrome-conjugated antibodies directed against CD31 and fluorochrome-conjugated annexin-V were used to assess the quantities of CMFDA^+^ MPs (left panel), of CD31^+^ MPs (middle panel), of annexin-V^+^ MPs (right panel) and were compared to the untreated conditions (dotted line). Data are mean ± SEM of 5 independent experiments presented as % of untreated (control) (B) MPs from mouse EOMA cells labeled with the CMFDA cell tracker were incubated 1 and 6 hours at 37°c, in absence or in presence of indicated concentrations of mouse recombinant sPLA_2_ IIA, V, X, or 1µg/ml of the inactive mutant X H48Q. Fluorochrome-conjugated antibodies directed against CD31 and fluorochrome-conjugated annexin-V were used to determine the concentrations of CMFDA^+^ MPs (left panel), of CD31^+^ MPs (middle panel), of annexin-V^+^ MPs (right panel) and then compared to the untreated conditions (dotted line). Data are mean ± SEM of 5 independent experiments presented as % of untreated (control). # P< .01; § P< .001.

We observed that none of the sPLA_2_s tested could impact the detection of endothelial cell MPs when an intracellular dye or antibodies against the CD31 were used (**Fig. 5A–B left and middle panel**). However, all the sPLAs tested induced a significant drop in annexin-V^+^ MPs, through PS hydrolysis (**Fig. 5A–B right panel**). Together, these results suggest that sPLA_2_s cannot clear endothelial cell-derived MPs but can use the exposed PS as substrate, thereby interfering in their detection through annexin-V.

### Impact of sPLA_2_s on MPs from apoptotic thymocytes

We next aimed to verify whether sPLA_2_s could use apoptotic cell-derived MPs as substrate, thereby impacting their detection and quantification. The thymus is a central immune organ where the positive and negative selection of T lymphocytes takes place [71]. During this selection process, the majority of the thymocytes are eliminated by apoptosis (about 95–97% of thymocytes die by apoptosis) [72]. Thymocytes, like other apoptotic cells, release MPs [73]. Moreover, sPLA_2_ IIA, V and X are expressed in the thymus [74, 75]. Thus, we addressed the effect of sPLA_2_s on human and mouse thymocyte MPs.

**Figure 6:**
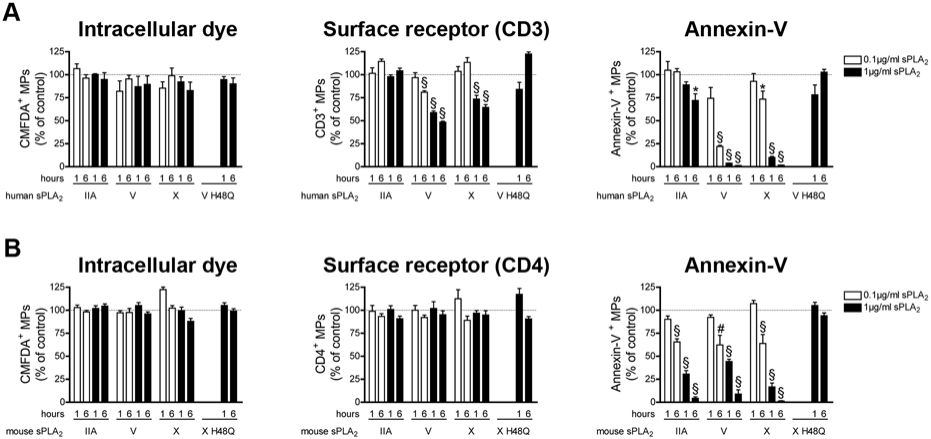
Impact of sPLA_2_s on MPs from apoptotic thymocytes. (A) MPs from human apoptotic thymocytes labeled with the CMFDA cell tracker were incubated for 1 and 6 hours at 37°c in absence or in presence of indicated concentrations of human recombinant sPLA_2_ IIA, V, X, or 1µg/ml of the inactive mutant V H48Q. Fluorochrome-conjugated antibodies directed against CD3 and fluorochrome-conjugated annexin-V were used to assess the quantities of CMFDA^+^ MPs (left panel), of CD3^+^ MPs (middle panel), of annexin-V^+^ MPs (right panel) and were compared to the untreated conditions (dotted line). Data are mean ± SEM of 5 independent experiments presented as % of untreated (control) (B) MPs from mouse apoptotic thymocytes labeled with the CMFDA cell tracker were incubated 1 and 6 hours at 37°c, in absence or in presence of indicated concentrations of mouse recombinant sPLA_2_ IIA, V, X, or 1µg/ml of the inactive mutant X H48Q. Fluorochrome-conjugated antibodies directed against CD4 and fluorochrome-conjugated annexin-V were used to determine the concentrations of CMFDA^+^ MPs (left panel), of CD4^+^ MPs (middle panel), of annexin-V^+^ MPs (right panel) and then compared to the untreated conditions (dotted line). Data are mean ± SEM of 5 independent experiments presented as % of untreated (control). * P< .05; # P< .01; § P< .001.

Using apoptotic thymocytes isolated from the thymus of human newborns that underwent thymectomies, we found that human sPLA_2_ V and X efficiently reduced the number of annexin-V^+^ MPs, CD3^+^ MPs (**Fig. 6A middle and right panel**) and CD4^+^ MPs (**S7 Fig**) through the sPLA_2_ catalytic activity, pointing to a potential role of these sPLA_2_s in clearance of human thymocyte MPs. sPLA_2_ IIA had only a modest, but significant, impact on the number of annexin-V^+^ MPs. None of the sPLA_2_s tested could impact the detection of MPs when the intracellular dye CMFDA was used (**Fig. 6A left panel**).

We made different observations in mice. We found that none of the mouse sPLA_2_s had an effect on detection of mouse thymocyte MPs when an intracellular dye or an antibody against surface antigen was used (**Fig. 6B left and middle panel**). Furthermore, we observed that all the murine sPLA_2_s tested were highly potent at hydrolyzing PS, thereby interfering in the quantification of murine annexin-V^+^ thymocyte MPs (**Fig. 6B right panel**).

Taken together, our data suggest that human sPLA_2_ V and X, but not the murine enzymes, can efficiently clear human thymocyte MPs. Our results also demonstrate that all the sPLA_2_s tested can efficiently hydrolyze PS on surface of apoptotic thymocyte MPs.

### Impact of sPLA_2_s on MPs from the male reproductive tract

In the epididymal fluid, MPs called epididymosomes convey microRNAs and play key roles in post-testicular maturation of spermatozoa [17, 60]. Moreover, sPLA_2_ IIA, V and X are also expressed in this fluid where they contribute to sperm maturation [47, 76, 77]. We thus determined the impact of sPLA_2_s on the detection of MPs present in the epididymal fluid.

**Figure 7:**
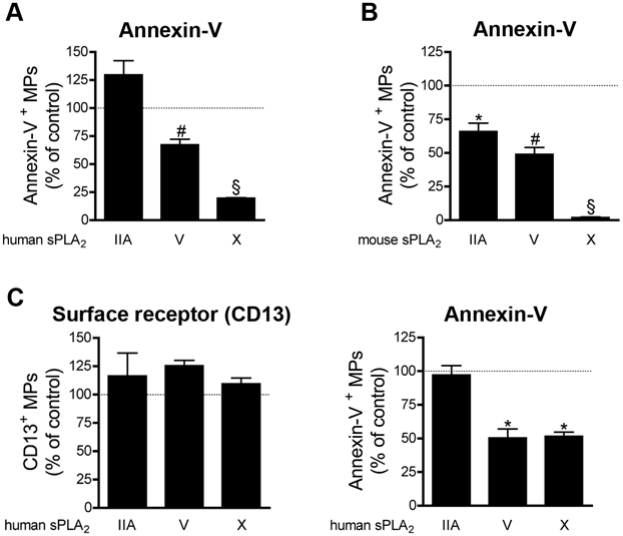
Impact of sPLA_2_s on MPs from the male reproductive tract. (A) Human epididymosomes were incubated 6 hours at 37°c in absence or in presence of 1µg/ml of human recombinant sPLA_2_ IIA, V, X. Fluorochrome-conjugated annexin-V was used to assess the quantities of annexin-V^+^ MPs and were compared to the untreated conditions (dotted line). Data are mean ± SEM of 3 independent experiments presented as % of untreated (control). (B) Mouse epididymosomes were incubated 6 hours at 37°c in absence or in presence of 1µg/ml of mouse recombinant sPLA_2_ IIA, V, X. Fluorochrome-conjugated annexin-V was used to assess the quantities of annexin-V^+^ MPs and were compared to the untreated conditions (dotted line). Data are mean ± SEM of 4 independent experiments presented as % of untreated (control). (C) Human prostasomes were incubated 6 hours at 37°c in absence or in presence of 1µg/ml of human recombinant sPLA_2_ IIA, V, X. Fluorochrome-conjugated antibodies directed against CD13 and fluorochrome-conjugated annexin-V were used to determine the concentrations of CD13^+^ MPs (left panel), of annexin-V^+^ MPs (right panel) and were compared to the untreated conditions (dotted line). Data are mean ± SEM of 4 independent experiments presented as % of untreated (control) * P< .05; # P< .01; § P< .001.

For these studies, we used the endogenous MPs present in epididymal fluid, and thus the intracellular dye could not be included to our assays. Furthermore, the markers CD9 and CD63 (detected in epididymosomes by proteomic approaches) did not give satisfying results and, despite numerous tests, we failed to identify a specific surface marker for epididymosomes. Given the limited amount of material available and the aforementioned reasons, we focused our attention on the impact of the various sPLA_2_s on MP detection via annexin-V. We found that all the sPLA_2_s tested, except human sPLA_2_ IIA, could decrease the number of annexin-V^+^ MPs. Murine and human sPLA_2_ X were by far the most potent enzymes at hydrolyzing the PS on epididymosomes (**Fig. 7A–B**).

We also tested the impact of sPLA_2_s on another type of MPs present in seminal fluid: the prostasomes. Prostasomes are MPs released by the prostate and are involved in sperm motility and acrosome reaction [78]. We observed that in presence of sPLA_2_ V and X, but not IIA, the quantities of annexin-V^+^ MPs detected decreased (**Fig. 7C right panel**). When an antibody against the aminopeptidase expressed on prostasomes (CD13) was used [79, 80], no decrease of CD13^+^ prostasomes was observed (**Fig. 7C left panel**), suggesting that prostasomes are not cleared by these sPLA_2_s. Thus, we demonstrate that sPLA_2_ V and X efficiently hydrolyze the PS on MPs present in the seminal fluid.

## Discussion

The interest toward the understanding of MP functions in biology and usage of MPs as potent biomarkers is growing rapidly. Hence, it becomes necessary to improve the methods of detection of MPs and to comprehend the factor(s) that might impede their detection. Our observations validate high sensitivity flow cytometry for the detection of MPs derived from various cellular lineages. Furthermore, we shed light on the impact of sPLA_2_s, which are concomitantly expressed with MPs in biological fluids, on quantification and detection of microparticles. We demonstrate that sPLA_2_ enzymes can use MPs as substrate and thereby might impair their quantification if expressed in sufficient concentrations. Importantly, this action by sPLA_2_ depends on the sPLA_2_ groups implicated, the species studied (human or mouse), the cellular source of MPs and the means of detection employed (annexin-V or antigen recognition by antibodies) (recapitulated in **Table 1**).

**Table 1:** Impact of human and mouse sPLA_2_s on detection of MPs from different cell origins.

	**Species**	**Mean of detection**	**sPLA_2_ IIA**	**sPLA_2_ V**	**sPLA_2_ X**
Platelets	Human (Collagen)	Intracellular tracker	-	-	-
		Antibody (CD41)	-	-	-
		Annexin-V	-	+	+
	Human (Thrombin)	Intracellular tracker	-	-	-
		Antibody (CD41)	-	-	-
		Annexin-V	-	+	+
	Human (HA-IgG)	Intracellular tracker	-	-	-
		Antibody (CD41)	-	-	-
		Annexin-V	-	+	+
	Mouse (Collagen)	Intracellular tracker	-	-	-
		Antibody (CD41)	-	-	-
		Annexin-V	++	++	++
Erythrocytes	Human	Antibody (CD235a)	-	-	-
		Annexin-V	-	+	++
	Mouse	Antibody (TER-119)	-	-	-
		Annexin-V	+	-	+
Endothelial cells	Human	Intracellular tracker	-	-	-
		Antibody (CD31)	-	-	-
		Annexin-V	+	+++	++
	Mouse	Intracellular tracker	-	-	-
		Antibody (CD31)	-	-	-
		Annexin-V	+++	++	++
Thymocytes	Human	Intracellular tracker	-	-	-
		Antibody (CD3)	-	+	+
		Annexin-V	+	++	+
	Mouse	Intracellular tracker	-	-	-
		Antibody (CD4)	-	-	-
		Annexin-V	++	++	++
Epididymosomes	Human	Annexin-V	-	+	+
	Mouse	Annexin-V	+	+	+
Prostasomes	Human	Antibody (CD13)	-	-	-
		Annexin-V	-	+	+

(-) No significant impact on detection

(+) Significant impact on detection

(++) Impact on detection within 6h at 0.1µg/ml and within 1h at 1µg/ml (≤ 75% of control and ≤ 50% of control, respectively)

(+++) Important impact on detection within 1h at 0.1µg/ml (≤ 75% of control)

The use of artificial microspheres for size calibration of the FSC remains imperfect as beads and cellular MPs display different refractive index [18, 19]. Notably, it was demonstrated that depending on type of flow cytometer used, beads are differently resolved by the FSC [20, 27, 28, 81, 82]. In this present study, we used high sensitivity flow cytometry combined with polystyrene beads for the creation of a MP gate. This approach was successfully used to resolve, on the FSC-PMT axis, MPs from organelle-containing MPs and MPs decorated with autoantibodies [12, 22]. Although this approach can be useful as reference particles to aid in the standardization of instrument setup, we acknowledge its potential limitations, as it cannot determine the size of MPs. The development of calibration vesicles having a refractive index similar to cellular MPs is needed to improve the detection and thus the comprehension of MP physio(patho)logical functions.

Mammalian sPLA_2_s have been described nearly 2 decades ago. They efficiently utilize RBC, platelet and whole blood cell MPs as substrate from which they release lysophospholipids [36]. Accordingly, several investigators prudently interpreted their MP quantifications, which might have been altered by the presence of sPLA_2_s [15, 16, 51, 53, 54, 55, 56, 57, 58]. Given that sPLA_2_s are overexpressed in the synovial fluid of RA patients, pioneer investigations suggested that annexin-V probes could not be used to detect MPs in these conditions [52]. However, no studies had formally assessed whether sPLA_2_s could actually interfere in the detection of MPs. Surprisingly, our observations demonstrate that, to precisely assess MP quantifications, the cellular origin of the MPs measured and the identity/concentration of the sPLA_2_ group(s) present both have to be considered. The co-expression in RA synovial fluid of sPLA_2_ IIA [43] and of platelet MPs [14], which were previously efficiently detected using annexin-V conjugated probes [12, 14, 29, 30], prompted our evaluation of MPs and sPLA_2_-IIA in these conditions. Consistent with our observations, which revealed that the detection of platelet MPs is unaffected by sPLA_2_ IIA, we confirmed that annexin-V can be efficiently utilized in body fluids rich in sPLA_2_ IIA when the concentration of platelet MPs is assessed. Nonetheless, whether sPLA_2_s might impact MP production and half-life, and thereby indirectly affect MP concentrations *in vivo* remains to be established.

Based on our observations, we recommend using a combination of at least two different markers of MPs for flow cytometric analyses (*i.e.* annexin-V, surface antigens, commercial dyes and transgenic expression of fluorescent proteins). Indeed, sPLA_2_s occasionally interfered in MP quantifications based solely on annexin-V, while the surface antigen detection using antibodies and the dyes were rather resistant, suggesting that sPLA_2_ enzymes, even at high concentrations, do not consume MPs. The only exception is the MPs originating from apoptotic thymocytes, which appeared to be cleared by sPLA_2_ V and X as PS, CD3 and CD4 were eliminated from MP surface. These findings further suggest that a physiological function of sPLA_2_ (group V and X) might be the clearance of the apoptotic thymocyte MPs that are constantly produced in the thymus during the selection of mature T cells.

Depending on their origin, all the MPs were not equally susceptible to sPLA_2_ treatments, which points to specific regulatory mechanisms expressed by MPs themselves. We also hypothesize that the stimulus behind the generation of MPs might have an impact on MP susceptibility to sPLA_2_. Indeed, as different platelet stimuli induce distinct MP protein content, we speculate that the surface lipid composition might also differ depending on the MP trigger [64]. Similarly, depending on their group (sPLA_2_ IIA, V or X) and species of origin (mouse vs human), sPLA_2_s displayed distinct activities on MPs. These observations support the notion that the different sPLA_2_ groups are not isozymes and might play non-redundant biological roles, and suggest that the murine and human sPLA_2_s might not necessarily be orthologous enzymes. It is important to note that rather high concentrations of sPLA_2_ (1µg/ml) were sometimes needed to impact MP detection. The concentration of sPLA_2_s in diverse biological fluids such as blood, tears, synovial and seminal fluids has been previously reported [43, 83, 84, 85, 86]. In these studies, sPLA_2_ IIA was the only enzyme assayed and its concentration ranged from 0.001 to 15 µg/ml. These concentrations are rarely present *in vivo*, and have been detected so far only in the blood of septic patients, in synovial fluid of RA patients, in tears and seminal fluids [43, 84, 85, 87]. More studies are thus needed to specifically determine the concentrations of the different sPLA_2_ groups in biological fluids in healthy and pathological conditions and to determine the actual significance of the impact of sPLA_2_s on MP assessment.

The hydrolysis of PS by certain sPLA_2_ groups might provide an explanation for the annexin-V negative MPs present in plasma visualized using cryo-electron microscopy [9, 10, 11, 12]. Furthermore, this action might be highly relevant in the biology of MPs. Indeed, studies have demonstrated the importance of PS in the rapid clearance of MPs (<10 minutes to some hours) from blood circulation [4, 5, 6], notably through interactions with lactadherin and developmental endothelial locus-1 (Del-1) [7, 8]. The action of sPLA_2_s, through the hydrolysis of PS, might increase MP half-life in circulation, thereby allowing them to deliver their content (e.g. microRNA, mRNA, proteins) in recipient target cells [13, 88]. Furthermore, PS expressed by MPs is a well-recognized procoagulant factor capable of promoting the assembly of components of the clotting cascade [3, 89, 90]. Thus, sPLA_2_s-mediated PS hydrolysis might also regulate coagulation. In addition, the hydrolysis of phospholipids at the sn-2 position generates lysophospholipids and free fatty acids [31, 32], which can be metabolized into potent lipid mediators, relevant to several physio—and pathological conditions.

Precise detection and quantification of MPs in biological fluids and cell supernatants is crucial for the utilization of MPs as biomarker and the understanding of their functions. Previous studies defined the most appropriate pre-analytical conditions for optimal isolation of MPs [64, 91, 92, 93]. Others demonstrated that protein aggregates could interfere in cytofluorometric analyses of MPs [29]. Herein, we reveal the impact of a family of enzymes co-expressed with MPs in diverse biological fluids and capable of potentially altering MP detection. Using the most recent approaches available in cytofluorometry our study provides precious information for the interpretation of MP quantifications and will contribute to the delineation of the functions of MPs in biology.

## Supporting Information

S1 Fig Detection of CMFDA^+^ human platelets MPs and YFP^+^ mouse platelets MPs using high sensitivity flow cytometry. (A) FSC-PMT and SSC portrayal of CMFDA^+^ platelet MPs from unlabeled (as control) and CMFDA^+^ human platelets. Total CMFDA^+^ particles are included in the green gate (left and middle panel) and the quantity of CMFDA^+^ MPs was determined in the CMFDA MP gate (right panel). Data are representative of 5 independent experiments. (B) FSC-PMT and SSC portrayal of YFP^+^ platelet MPs from unlabeled and YFP mouse platelets. Total YFP^+^ events are presented in the green gate (left and middle panel) and the quantity of YFP^+^ MPs was determined in the YFP MP gate (right panel). Data are representative of 5 independent experiments.
(TIF)

S2 Fig Detection of erythrocyte MPs and sensitivity to Triton and EDTA treatments. (A, B, C) FSC-PMT and SSC portrayal of erythrocyte MPs detected using annexin-V and an antibody against CD235a in absence of treatment (control) (A), and in presence of 0.05% Triton (B) and 50µM EDTA (C). Total annexin-V^+^ events are comprised in the pink gate (middle panel) and the quantity of annexin-V^+^ MPs was determined in the annexin-V MP gate (upper panel). Total CD235a^+^ events are presented in the green gate (middle panel) and the quantity of CD235a^+^ MPs was determined in the CD235a MP gate (lower panel). Data are representative of 5 independent experiments. (D) Triton sensitivity of the erythrocyte MPs detected using annexin-V (left panel) and anti-CD235a (right panel) presented as % of untreated (control). (E) EDTA sensitivity of annexin-V (left panel) and CD235a (right panel) labeling presented as % of untreated (control). Data are representative of 5 independent experiments.
(TIF)

S3 Fig Detection of HUVEC MPs and sensitivity to Triton and EDTA treatments. (A, B, C) FSC-PMT/SSC portrayal of HUVEC MPs detected with fluorochrome-conjugated annexin-V and antibody against CD31 in absence of treatment (control) (A), and treated with 0.05% triton (B) and 50µM EDTA (C). Total annexin-V^+^ events are included in the pink gate (middle panel) and the quantity of annexin-V^+^ MPs was determined in the annexin-V MP gate (upper panel). Total CD31^+^ events are included in the orange gate (middle panel) and the quantity of CD31^+^ MPs was determined in the CD31 MP gate (lower panel). Data are representative of 5 independent experiments. (D) Triton sensitivity of the HUVEC MPs detected using annexin-V (left panel) and anti-CD31 (right panel) presented as % of untreated (control). (E) EDTA sensitivity of annexin-V (left panel) and CD31 (right panel) labeling presented as % of untreated (control). Data are representative of 5 independent experiments. (F) Portrayal of CMFDA^+^ HUVEC MPs and MPs from unlabeled HUVEC. Total CMFDA^+^ events are included in the green gate (left and middle panel) and the quantity of CMFDA^+^ MPs was determined in the CMFDA MP gate (right panel). Data are representative of 5 independent experiments.
(TIF)

S4 Fig Detection of apoptotic thymocyte MPs and sensitivity to Triton and EDTA treatments. (A, B, C) Portrayal of human apoptotic thymocyte MPs detected with fluorochrome-conjugated annexin-V and antibody against CD3 in absence of treatment (control) (A), and treated with 0.05% triton (B) and 50µM EDTA (C). Total annexin-V^+^ events are included in the pink gate (middle panel) and the quantity of annexin-V^+^ MPs was determined in the Annexin-V MP gate (upper panel). Total CD3^+^ events are detected in the red gate (middle panel) and the quantity of CD3^+^ MPs is determined in the CD3 MP gate (lower panel). Data are representative of 5 independent experiments. (D) Triton sensitivity of the human apoptotic thymocyte MPs detected using annexin-V (left panel) and anti-CD3 (right panel) presented as % of untreated (control). (E) EDTA sensitivity of annexin-V (left panel) and CD3 (right panel) labeling presented as % of untreated (control). Data are representative of 5 independent experiments. (F) Portrayal of CMFDA^+^ thymocyte MPs from unlabeled and CMFDA-labeled thymocyte. Total CMFDA^+^ events are presented in the green gate (left and middle panel) and the quantity of CMFDA^+^ MPs was determined in the CMFDA MP gate (right panel). Data are representative of 5 independent experiments.
(TIF)

S5 Fig Detection of epididymosomes and detergent treatment. (A, B) FSC-PMT/SSC portrayal of human epididymosomes detected with fluorochrome-conjugated annexin-V in absence of treatment (control) (A), and treated with 0.05% triton (B). Total annexin-V^+^ events are comprised in the pink gate (middle panel) and the quantity of annexin-V^+^ MPs was determined in the Annexin-V MP gate (upper panel). Data are representative of 3 independent experiments. (C) Triton sensitivity of the human epididymosomes detected using fluorochrome-conjugated annexin-V presented as % of untreated (control). Data are representative of 3 independent experiments
(TIF)

S6 Fig Enzymatic activities of human sPLA_2_ IIA, V and X on *E. Coli* membranes. The assays of human sPLA_2_s enzymatic activities were carried out using [^3^H]-oleic acid radiolabeled E. coli membranes. After incubation with sPLA_2_s, the supernatant containing released radiolabeled oleate was submitted to scintillation counting.
(TIF)

S7 Fig Impact of human sPLA_2_s on CD4^+^ MPs from human apoptotic thymocytes. MPs from human apoptotic thymocytes were incubated for 1 hours at 37°c in absence or in presence of 1µg/ml of human recombinant sPLA_2_ IIA, V, X and V H48Q (inactive mutant). Fluorochrome-conjugated antibodies against CD4 were used to assess the quantities of CD4^+^ MPs and were compared to the untreated conditions (dotted line). Data are mean ± SEM of 5 independent experiments presented as % of untreated (control). § P< .001.
(TIF)
