# Supplementary figures and images for: Detection and Quantification of Microparticles from Different Cellular Lineages Using Flow Cytometry. Evaluation of the Impact of Secreted Phospholipase A2 on Microparticle Assessment
Source: PLoS One. 2015 Jan 14;10(1):e0116812. doi: 10.1371/journal.pone.0116812 (PMC4294685; doi:10.1371/journal.pone.0116812)

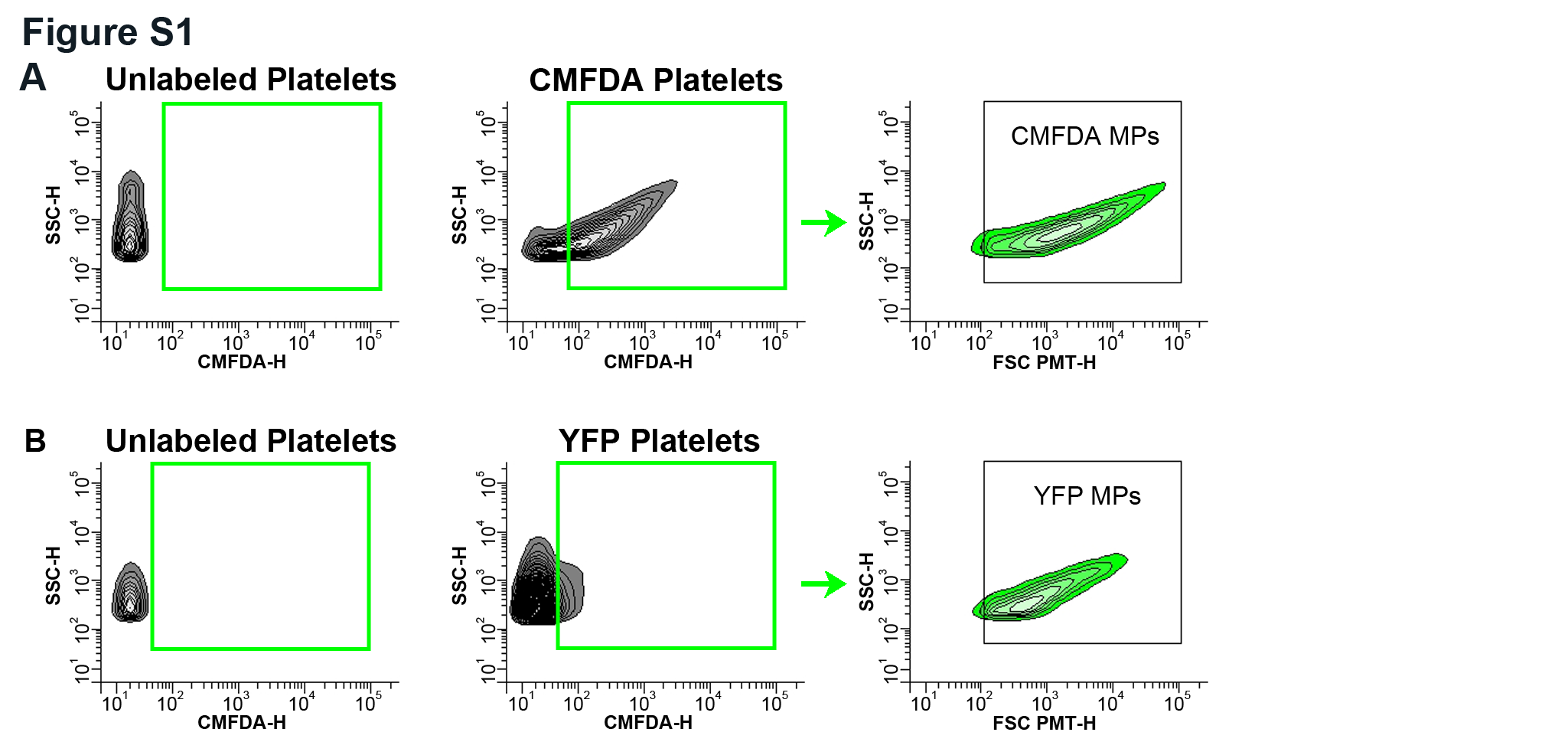

Supplement: S1 Fig — (A) FSC-PMT and SSC portrayal of CMFDA+ platelet MPs from unlabeled (as control) and CMFDA+ human platelets. Total CMFDA+ particles are included in the green gate (left and middle panel) and the quantity of CMFDA+ MPs was determined in the CMFDA MP gate (right panel). Data are representative of 5 independent experiments. (B) FSC-PMT and SSC portrayal of YFP+ platelet MPs from unlabeled and YFP mouse platelets. Total YFP+ events are presented in the green gate (left and middle panel) and the quantity of YFP+ MPs was determined in the YFP MP gate (right panel). Data are representative of 5 independent experiments. (TIF) [file pone.0116812.s001.tif]

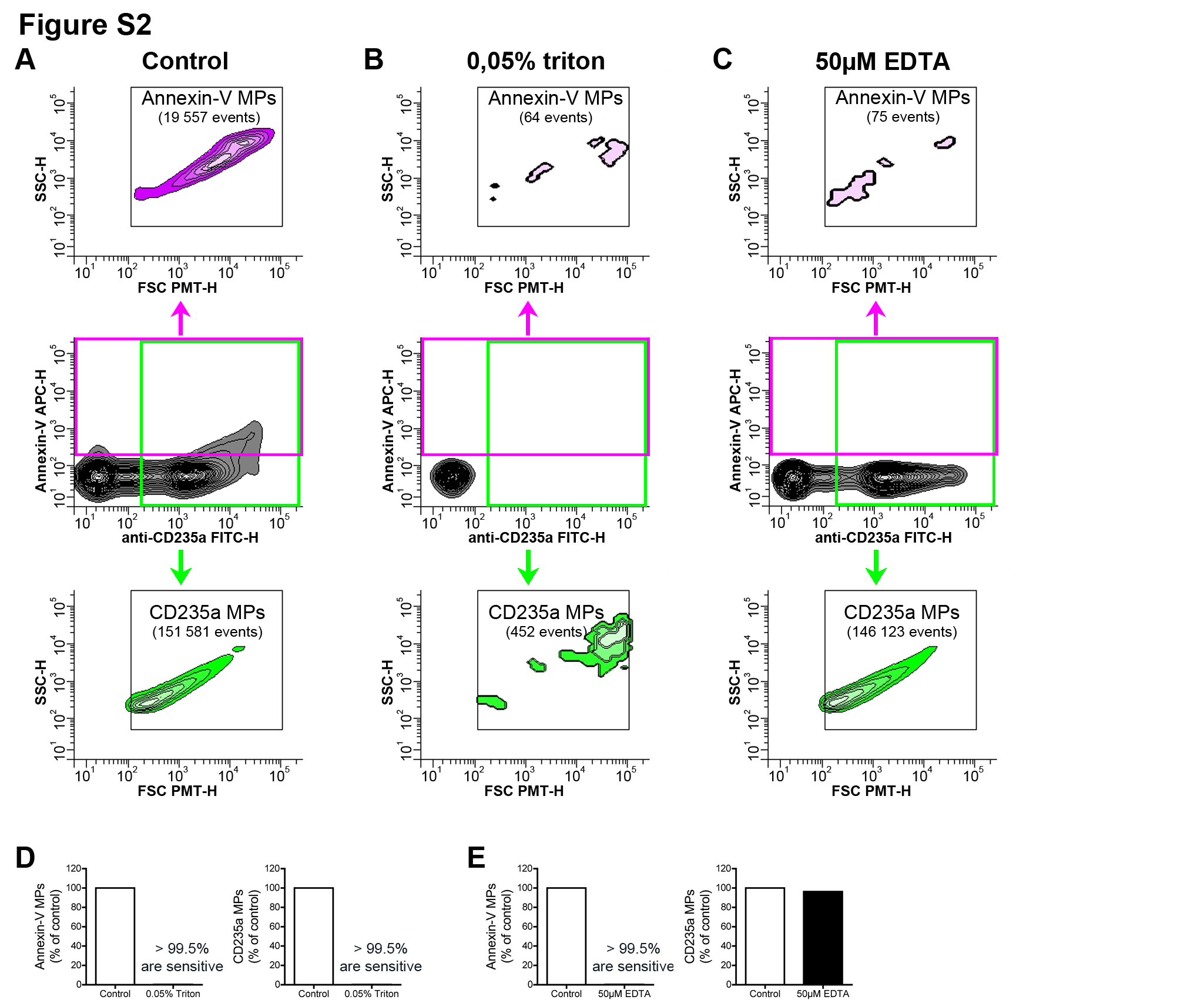

Supplement: S2 Fig — (A, B, C) FSC-PMT and SSC portrayal of erythrocyte MPs detected using annexin-V and an antibody against CD235a in absence of treatment (control) (A), and in presence of 0.05% Triton (B) and 50µM EDTA (C). Total annexin-V+ events are comprised in the pink gate (middle panel) and the quantity of annexin-V+ MPs was determined in the annexin-V MP gate (upper panel). Total CD235a+ events are presented in the green gate (middle panel) and the quantity of CD235a+ MPs was determined in the CD235a MP gate (lower panel). Data are representative of 5 independent experiments. (D) Triton sensitivity of the erythrocyte MPs detected using annexin-V (left panel) and anti-CD235a (right panel) presented as % of untreated (control). (E) EDTA sensitivity of annexin-V (left panel) and CD235a (right panel) labeling presented as % of untreated (control). Data are representative of 5 independent experiments. (TIF) [file pone.0116812.s002.tif]

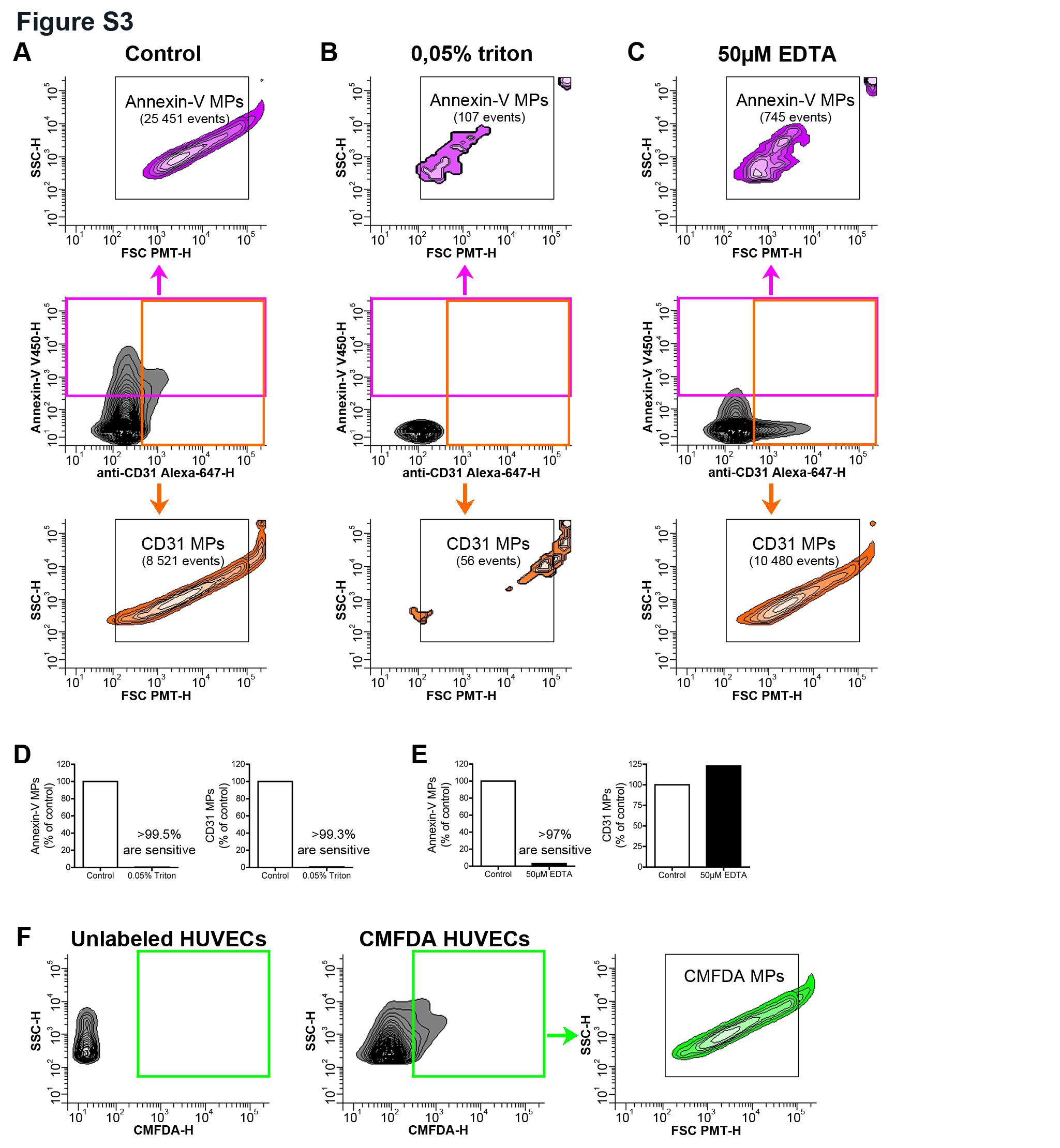

Supplement: S3 Fig — (A, B, C) FSC-PMT/SSC portrayal of HUVEC MPs detected with fluorochrome-conjugated annexin-V and antibody against CD31 in absence of treatment (control) (A), and treated with 0.05% triton (B) and 50µM EDTA (C). Total annexin-V+ events are included in the pink gate (middle panel) and the quantity of annexin-V+ MPs was determined in the annexin-V MP gate (upper panel). Total CD31+ events are included in the orange gate (middle panel) and the quantity of CD31+ MPs was determined in the CD31 MP gate (lower panel). Data are representative of 5 independent experiments. (D) Triton sensitivity of the HUVEC MPs detected using annexin-V (left panel) and anti-CD31 (right panel) presented as % of untreated (control). (E) EDTA sensitivity of annexin-V (left panel) and CD31 (right panel) labeling presented as % of untreated (control). Data are representative of 5 independent experiments. (F) Portrayal of CMFDA+ HUVEC MPs and MPs from unlabeled HUVEC. Total CMFDA+ events are included in the green gate (left and middle panel) and the quantity of CMFDA+ MPs was determined in the CMFDA MP gate (right panel). Data are representative of 5 independent experiments. (TIF) [file pone.0116812.s003.tif]

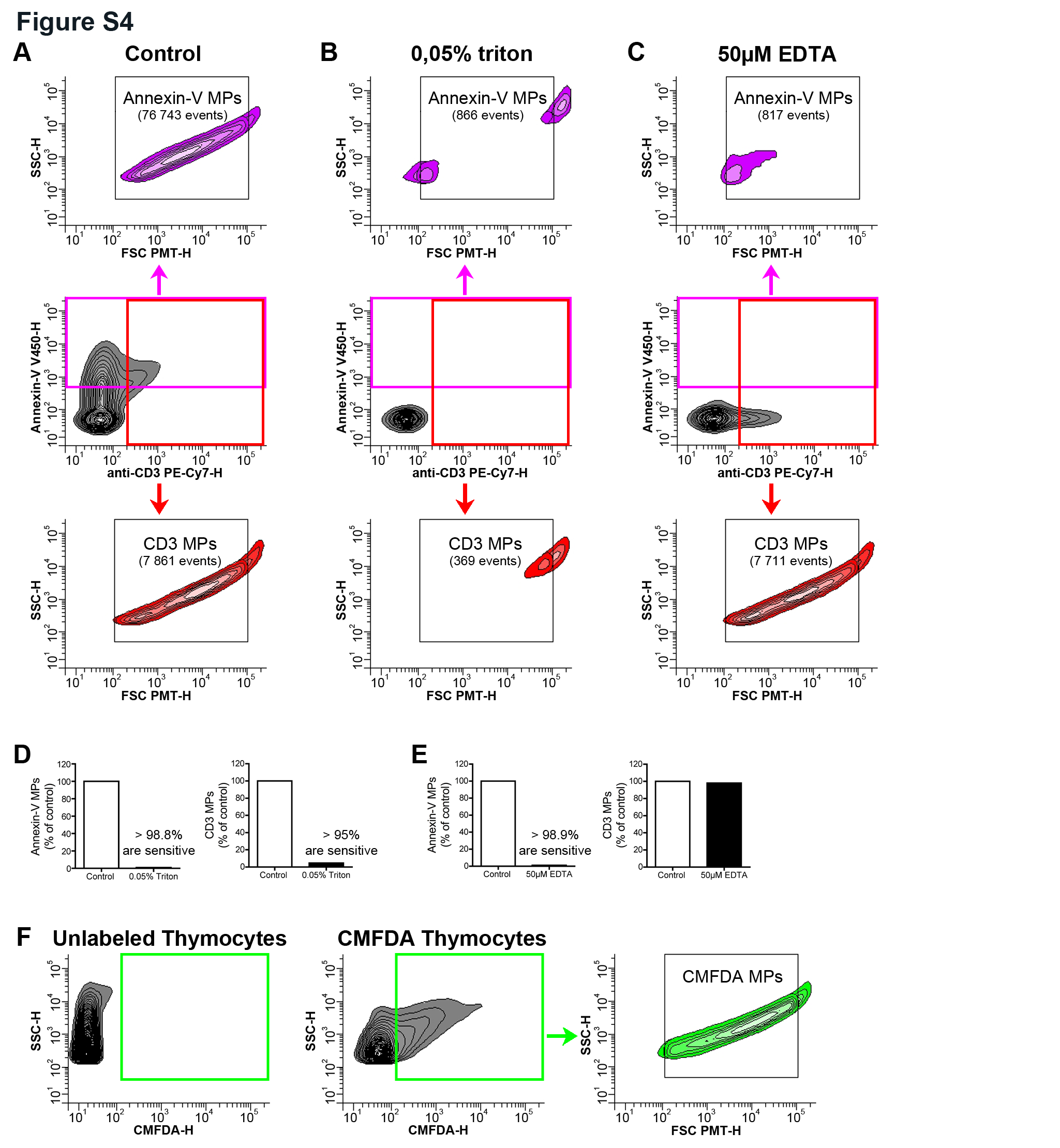

Supplement: S4 Fig — (A, B, C) Portrayal of human apoptotic thymocyte MPs detected with fluorochrome-conjugated annexin-V and antibody against CD3 in absence of treatment (control) (A), and treated with 0.05% triton (B) and 50µM EDTA (C). Total annexin-V+ events are included in the pink gate (middle panel) and the quantity of annexin-V+ MPs was determined in the Annexin-V MP gate (upper panel). Total CD3+ events are detected in the red gate (middle panel) and the quantity of CD3+ MPs is determined in the CD3 MP gate (lower panel). Data are representative of 5 independent experiments. (D) Triton sensitivity of the human apoptotic thymocyte MPs detected using annexin-V (left panel) and anti-CD3 (right panel) presented as % of untreated (control). (E) EDTA sensitivity of annexin-V (left panel) and CD3 (right panel) labeling presented as % of untreated (control). Data are representative of 5 independent experiments. (F) Portrayal of CMFDA+ thymocyte MPs from unlabeled and CMFDA-labeled thymocyte. Total CMFDA+ events are presented in the green gate (left and middle panel) and the quantity of CMFDA+ MPs was determined in the CMFDA MP gate (right panel). Data are representative of 5 independent experiments. (TIF) [file pone.0116812.s004.tif]

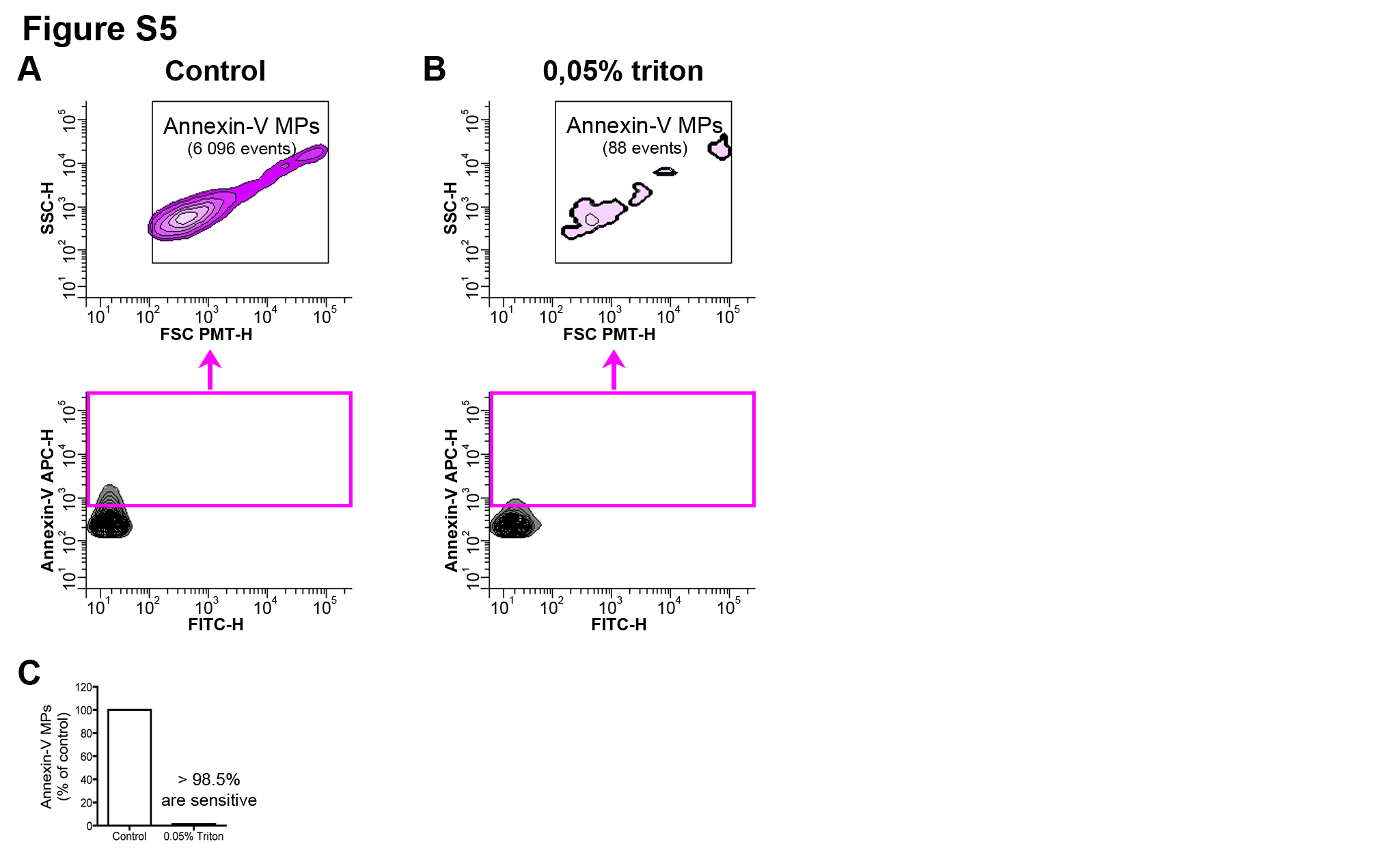

Supplement: S5 Fig — (A, B) FSC-PMT/SSC portrayal of human epididymosomes detected with fluorochrome-conjugated annexin-V in absence of treatment (control) (A), and treated with 0.05% triton (B). Total annexin-V+ events are comprised in the pink gate (middle panel) and the quantity of annexin-V+ MPs was determined in the Annexin-V MP gate (upper panel). Data are representative of 3 independent experiments. (C) Triton sensitivity of the human epididymosomes detected using fluorochrome-conjugated annexin-V presented as % of untreated (control). Data are representative of 3 independent experiments (TIF) [file pone.0116812.s005.tif]

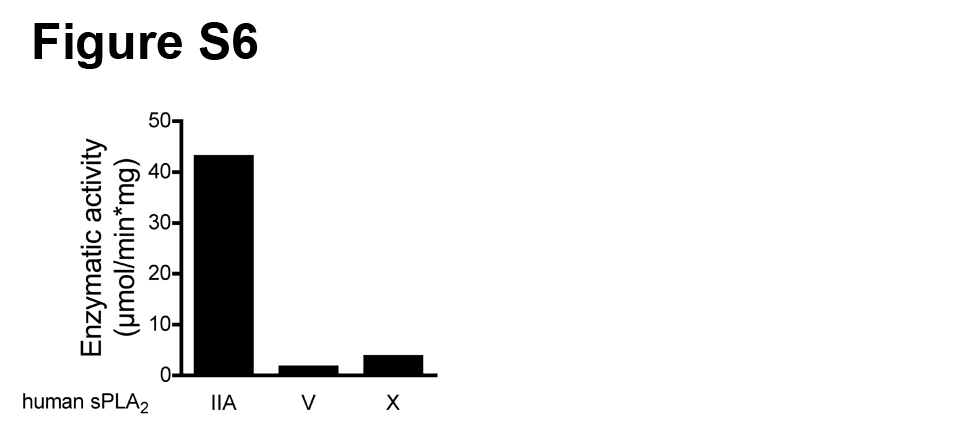

Supplement: S6 Fig — The assays of human sPLA2s enzymatic activities were carried out using [3H]-oleic acid radiolabeled E. coli membranes. After incubation with sPLA2s, the supernatant containing released radiolabeled oleate was submitted to scintillation counting. (TIF) [file pone.0116812.s006.tif]

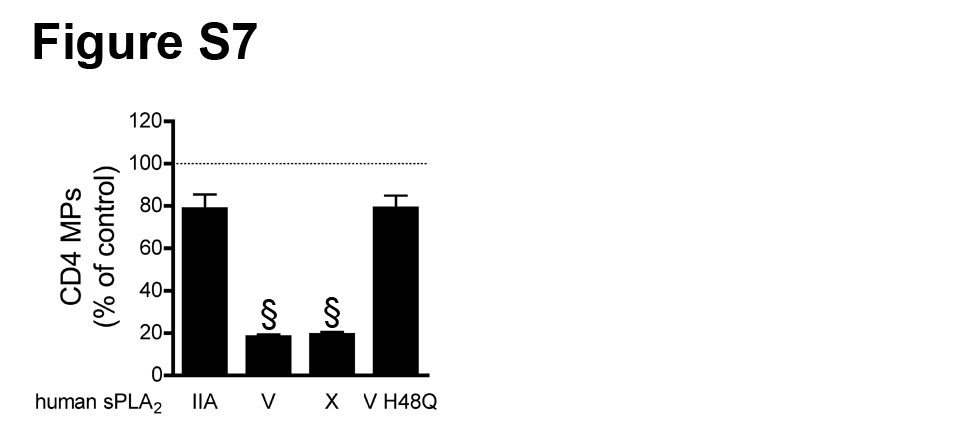

Supplement: S7 Fig — MPs from human apoptotic thymocytes were incubated for 1 hours at 37°c in absence or in presence of 1µg/ml of human recombinant sPLA2 IIA, V, X and V H48Q (inactive mutant). Fluorochrome-conjugated antibodies against CD4 were used to assess the quantities of CD4+ MPs and were compared to the untreated conditions (dotted line). Data are mean ± SEM of 5 independent experiments presented as % of untreated (control). § P< .001. (TIF) [file pone.0116812.s007.tif]
